# Cell type-specific Ca^2+^ signals govern mouse seminiferous tubule physiology

**DOI:** 10.1371/journal.pbio.3003910

**Published:** 2026-07-24

**Authors:** Justine A. Fischoeder, David Fleck, Jerome Schröer, Christopher Wiesbrock, Lina Kenzler, Christoph Weber-Hamacher, Ilian Schröder, Melissa Franke, Stefanie Kurth, Martin Strauch, Guiscard Seebohm, Dorit Merhof, Johannes Stegmaier, Naofumi Uesaka, Jennifer Spehr, Marc Spehr

**Affiliations:** 1 Department of Chemosensation, Institute for Biology II, RWTH Aachen University, Aachen, Germany; 2 Research Training Group 2416 MultiSenses – MultiScales, RWTH Aachen University, Aachen, Germany; 3 Institute of Imaging and Computer Vision, RWTH Aachen University, Aachen, Germany; 4 Department of Cardiology and Angiology, University Hospital of Münster, Münster, Germany; 5 Center for Digital Medicine, Faculty of Mathematics and Natural Sciences, Heinrich Heine University Düsseldorf, Düsseldorf, Germany; 6 Department of Cognitive Neurobiology, Graduate School of Medical and Dental Sciences, Institute of Science Tokyo, Tokyo, Japan; National Institute of Environmental Health Sciences, UNITED STATES OF AMERICA

## Abstract

Spermatogenesis, the complex developmental process of male germ cell proliferation, differentiation, and maturation, is the basis of male fertility. In the seminiferous tubules of the testes, spermatozoa are constantly generated from spermatogonial stem cells through a stereotyped sequence of divisions. The basic physiological principles, however, that control seminiferous tubule function remain poorly, if at all, defined. Here, we address cell type-specific seminiferous tubule signaling *in vitro* and *in vivo*. By monitoring changes in cellular Ca^2+^ concentration at high spatiotemporal resolution, we show that the three cell types that build the seminiferous epithelium—Sertoli, peritubular, and germ cells—each display unique Ca^2+^ signaling patterns. We reveal the underlying mechanisms and demonstrate that Sertoli cell Ca^2+^ signals are under gonadotropin regulation. Together, our experimental findings provide insights into seminiferous tubule signaling, its mechanistic basis, and its endocrine control.

## Abstract 2

Seminiferous tubules of the testes play an important role in sperm production, but their regulation remain unclear. This study shows that all the cell types that constitute the seminiferous epithelium display unique calcium signalling patterns that are under endocrine control.

## Introduction

Spermatogenesis ranks among the most complex, yet least understood, developmental processes in postnatal life. Given the intricate anatomy and complex endo-/ paracrinology of the testis, the physiological principles that control male germ cell development in mammals are notoriously difficult to unravel. Accordingly, we lack a conceptual understanding of many basic signaling mechanisms that control seminiferous tubule function. The stratified seminiferous epithelium is composed of columnar Sertoli cells, each associated with ≥30 germ cells at different developmental stages [1]. Spermatogenesis progresses through coordinated cycles [2,3] in which germ cells undergo sequential stages of differentiation. In mice, each spermatogenic cycle comprises 12 stages [4], and completes with the release of immotile haploid spermatozoa into the lumen of the seminiferous tubule (spermiation).

Together with testicular peritubular cells (TPCs) that surround the seminiferous tubules, Sertoli cells provide a unique microenvironment critical for spermatogenesis. They form the spermatogonial stem cell niche [5], establish the blood-testis barrier (BTB) [6] to compartmentalize the tubule into basal and immune-privileged adluminal regions, respectively, and they control epithelial cyclicity [7]. Premeiotic spermatogonia comprise a heterogeneous population and reside along the seminiferous tubule basement membrane [8]. As type B spermatogonia, they detach and enter meiosis.

Bidirectional communication between Sertoli and developing germ cells balances spermatogonial self-renewal and differentiation, synchronizes stage transitions, and regulates BTB restructuring dynamics [9]. Endocrine control of spermatogenesis along the hypothalamic–pituitary–testicular axis functionally converges on Sertoli cells [10], which express both androgen and follicle-stimulating hormone (FSH) receptors [11]. FSH regulates Sertoli cell physiology during both fetal and postnatal life, whereas androgen receptor expression begins during puberty when luteinizing hormone (LH) raises the intratubular testosterone concentration [12].

Ionized calcium (Ca^2+^) is the most versatile cellular messenger that impacts nearly every aspect of cellular life [13,14]. The exact physiological effects exerted by this universal tool of signal transduction are largely determined by the unique spatiotemporal profile of any given Ca^2+^ signal. Its reliability, specificity and speed depend on (*i*) Ca^2+^ release and influx mechanisms, (*ii*) cytoplasmic buffers that limit Ca^2+^ diffusion, and (*iii*) extrusion and storage processes that restore resting conditions, which are typically maintained at levels of ∼100–150 nM [15,16]. The molecular mediators involved in orchestrating discrete Ca^2+^ responses have collectively been designated as the Ca^2+^ signaling ‘toolkit’ [16]. Key members include a multitude of Ca^2+^-permeable ion channels, Na^+^/Ca^2+^ exchangers, plasma membrane Ca^2+^ ATPases, the mitochondrial Ca^2+^ uniporter, and the sarco/endoplasmic reticulum Ca^2+^ pump [17–20]. Frequently, toolkit members are organized into supramolecular signaling complexes. The coordinated and spatially controlled activity of such signalosomes confers cell-type-specific Ca^2+^ fingerprints.

Ca^2+^ signals encode information in their amplitude, kinetics, spatial extent and, notably, their frequency [21–23]. On the temporal scale, signals range from submillisecond events that trigger presynaptic vesicle release [24] to sustained Ca^2+^ elevations that regulate gene transcription [25]. Spatially, fixed and mobile endogenous buffers limit Ca^2+^ diffusion and organize signalosomes into micro- and nanodomains [26,27]. In such limited cytosolic volumes, local Ca^2+^ concentrations reach up to 100 μM [28]. Prolonged signals can propagate throughout the cytoplasm as regenerative saltatory Ca^2+^ waves [29]. This way, initially local Ca^2+^ signals can spread as global waves and affect distant Ca^2+^-regulated processes.

Ca^2+^ signals have been measured *in situ* in TPCs [30] and germ cells [31], as well as in cultured Sertoli cells [10,32,33]. In both human and mouse TPCs, purinoceptor activation triggers coordinated Ca^2+^ signals that drive seminiferous tubule contractions and, consequently, luminal sperm transport [30,34]. Both FSH and testosterone as well as ATP increase Ca^2+^ within Sertoli cells [10,33]. Their polarized morphology and cytoplasmic compartmentalization introduce a unique landscape to orchestrate Ca^2+^-sensitive responses. In adult rat Sertoli cells, the ATP-dependent Ca^2+^ signal alters Sertoli cell estradiol production, enzyme activity, and secretory behavior [35–38]. The distinct patterns of endoplasmic reticulum (ER) cisternae [39] and the mitochondrial network [33] in Sertoli cells indicate that Ca^2+^ signals may play important roles during BTB remodeling [40]. Little, however, is known about Ca^2+^ signaling in germ cells. While synchronous spontaneous Ca^2+^ oscillations have been described in clusters of mouse germ cells *in situ*, other cells displayed asynchronous signals [31].

Here, we analyze cell type-specific Ca^2+^ signaling in mouse TPCs, Sertoli cells, and premeiotic spermatogonia both *in vitro* and *in vivo*. In acute seminiferous tubule slices, we report distinct activity patterns that vary between cell types and predominantly depend on IP_3_-mediated signaling pathways. Intravital multiphoton imaging confirms unique and cell type-specific Ca^2+^ signaling fingerprints. Sertoli cells, in particular, display dynamic and spatiotemporally distinct patterns of *in vivo* activity. The level of activity is cycle stage-dependent, with highly active areas predominantly found in tubules close to or during spermiation. While short-lived Ca^2+^ signals lack obvious higher-order orchestration, more persistent activity appears synchronized and under gonadotropin control.

## Results

### Cells in the seminiferous tubule display distinct types of spontaneous activity

**Fig 1:**
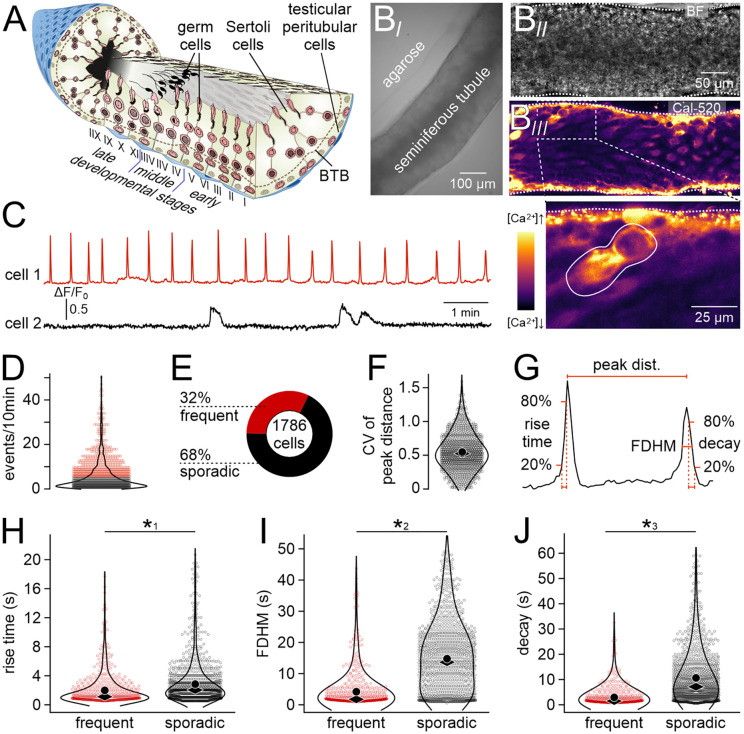
Cells in the seminiferous tubule display distinct types of spontaneous activity. **(A)** Schematic sketch of a mouse seminiferous tubule highlighting 12 stages (I–XII) of the spermatogenic cycle, which are arranged in consecutive order along the length of the tubule [3,30]. A single TPC layer (blue) lines the tubule. Columnar Sertoli cells, associated with numerous germ cells at different stages of development, span the tubule from basal lamina to lumen. The blood-testis barrier (BTB) separates the basal from the immune-privileged adluminal compartment. Spermatogenic cycle stages are categorized into early (I‒V), middle (VI–VIII), and late (IX‒XII) stages. **(B)** Brightfield (**B_*I&II*_**) and semi-confocal fluorescence (**B_*III*_**) micrographs of an acute mouse seminiferous tubule slice embedded in agarose and loaded with Cal-520/AM. Tubule walls indicated by dotted white lines, pseudocolors (inferno 256 color map) indicate relative Ca^2+^ concentration. A representative region depicting a transient Ca^2+^ elevation is highlighted (dashed white rectangle in (B_***III***_)). **(C)** Representative original traces (ΔF/F_0_, fluorescence intensity *vs.* time) illustrating two general types of spontaneous cellular activity (color code as in (D&E)). **(D)** Dot and violin plot illustrating Ca^2+^ signal count per cell over 10 min. Note the multimodal distribution with ≤ 5 events accounting for the largest data population (gray dots). **(E)** Wheel chart quantifying the occurrence of either activity pattern observed: sporadic (black; 1,215/1,786 cells) or frequent (red; 571/1,786 cells). **(F)** Dot and violin plot describing signal (ir)regularity as each cell’s inter-event interval CV (mean ± SD = 0.6 ± 0.3; median = 0.5). **(G–J)** Comparative analysis of signal kinetics. **(G)** Sketch depicting signal parameters analyzed. **(H–J)** Dot and violin plots comparing average rise time [(**H**); mean ± SD = 1.9 ± 2.3 s (red) *vs.* 2.8 ± 2.9 s (black), median = 1.1 s (red) *vs.* 2.0 s (black)], signal width [(**I**); mean ± SD = 4.1 ± 6.1 s (red) *vs.* 14.7 ± 11.2 s (black), median = 1.7 s (red) *vs.* 13.6 s (black)], and decay time [(**J**); mean ± SD = 2.7 ± 3.8 s (red) *vs.* 10.5 ± 10.5 s (black), median = 1.3 s (red) *vs.* 7.0 s (black)]. Activity patterns color-coded as in (D&E). Black dots represent mean, black diamonds display median values. Asterisks indicate statistical signiﬁcance (*p*^1^ < 0.0001; *p*^2^ < 0.0001; *p*^3^ < 0.0001; Mann–Whitney *U* test). BF, brightfield; BTB, blood-testis barrier; CV, coefficient of variation; FDHM, full duration at half maximum. The underlying numerical data for this figure is detailed in S1 Data.

Initially, we set out to describe spontaneous (i.e., not experimentally evoked) seminiferous tubule Ca^2+^ signals in a largely intact epithelial environment (Fig 1A). To this end, we bulk-loaded acute mouse seminiferous tubule slices (Fig 1B [30]) with the Ca^2+^ indicator Cal-520/AM. Prolonged (10 min) semi-confocal fluorescence imaging revealed essentially two types of spontaneous cellular activity (Fig 1C and S1 Movie). We categorize these patterns (Fig 1D and 1E; S1 Table) according to event number as (*i*) frequent activity (32% of cells; ≥ 6 events/10 min), or (*ii*) sporadic and seemingly random activity (sporadic; 68% of cells; ≤5 events/10 min). Analysis of inter-event interval coefficient of variation (CV; Fig 1F) reveals a broad, apparently normal distribution of individual CVs. The lack of a distinct cell population with low CV values suggests that rather few cells, if any, display highly periodic oscillatory signals.

Frequent and sporadic Ca^2+^ signal phenotypes differ in their kinetics (Fig 1G–1J). Frequently active cells display fast onset kinetics (Fig 1H) and relatively short durations (Fig 1I and 1J). By contrast, cells showing sporadic activity display longer lasting signals (Fig 1I and 1J) with significantly slower rise times (Fig 1H). Together, these data demonstrate that cells in the mouse seminiferous tubule essentially display either of two types of spontaneous activity. Cells either show fast rising and relatively brief Ca^2+^ transients that lack apparent periodicity, or signals are slower and more heterogeneous in duration.

### IP_3_-dependent signaling pathways dominate seminiferous tubule Ca^2+^ activity

**Fig 2:**
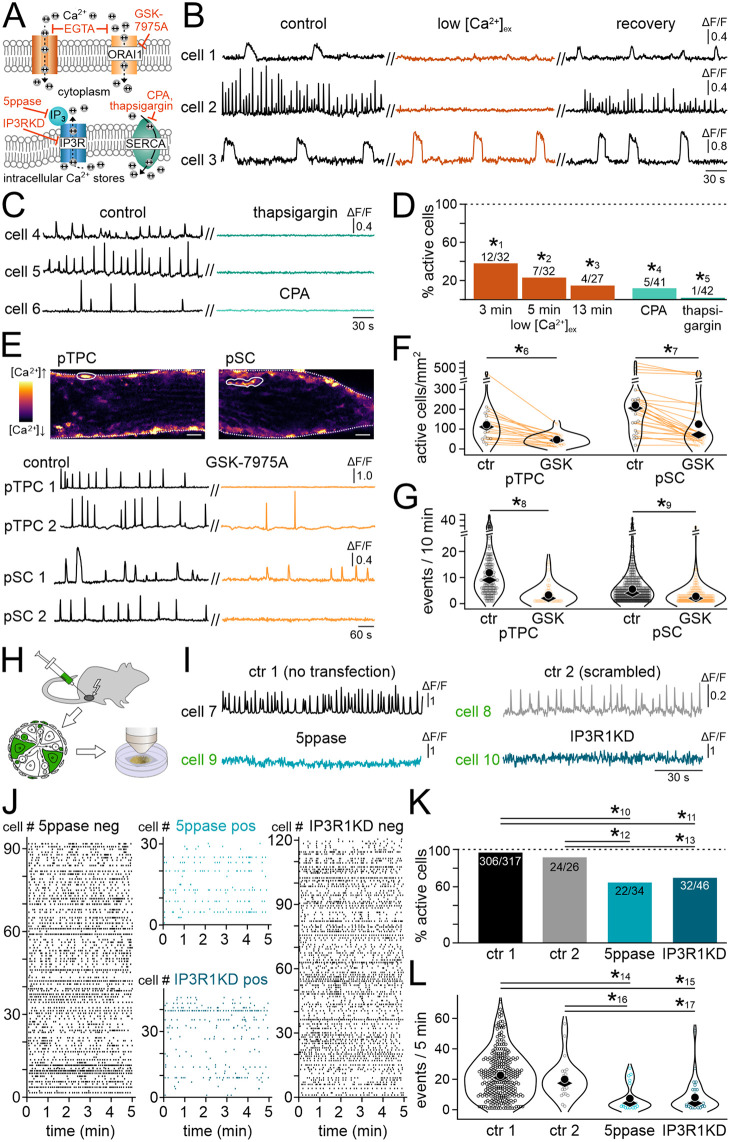
IP_3_-dependent signaling pathways dominate seminiferous tubule Ca^2+^ activity. **(A)** Diagram illustrating select members and mechanisms of the Ca^2+^ signaling toolkit. A Ca^2+^-permeable ion channel and the store-operated Ca^2+^ channel ORAI in the plasma membrane as well as the sarco/endoplasmic reticulum Ca^2+^ (SERCA) pump and an inositol 1,4,5-trisphosphate (IP_3_) receptor (IP3R) in the ER membrane are depicted. Experimental approaches to interfere with Ca^2+^ signaling are schematically shown in red, including extracellular Ca^2+^ chelation (EGTA), ORAI inhibition (GSK-7975A), IP3R knock-down (KD), IP_3_ hydrolysis by 5-phosphatase (5ppase), and Ca^2+^ depletion from the ER by SERCA inhibition (thapsigargin and cyclopiazonic acid (CPA)). **(B, C)** Original recordings from six representative cells show changes in Ca^2+^ concentration (ΔF/F_0_) over time. Traces depict signals prior to and after treatment with reduced extracellular Ca^2+^ (**B**) or thapsigargin / CPA **(C)**, respectively. **(D)** Quantification of residual cellular activity during treatment, derived from recordings as shown in (B, C). Asterisks indicate statistical signiﬁcance (*p*^1^–*p*^5^ < 0.0001; Fisher´s exact test). **(E)** Representative original recordings from putative TPCs (pTPCs) and Sertoli cells (pSCs). Traces depict fluorescence intensity (Δ*F*/*F*_0_) over time in absence (left; black) and presence (right; orange) of GSK-7975A (10 µM, 10 min preincubation). Top images show semi-confocal optical sections from isolated tubules. Tubule walls indicated by dotted white lines, pseudocolors (inferno color map) indicate relative Ca^2+^ concentration. A pTPC (left) and pSC (right) are categorized by their distinct shape and location, indicated as regions-of-interest (white lines). Scale bars: 50 µm. **(F, G)** Dot and violin plots quantifying activity before and during treatment, derived from paired recordings as shown in (E). **(F)** Active pTPCs (ctr: mean ± SD = 121.4 ± 85.5, median = 110; GSK: 46.5 ± 29.4, median = 43) or pSCs (ctr: mean ± SD = 220.3 ± 146.4, median = 205; GSK: mean ± SD = 124.6 ± 121.1, median = 71) per area (mm^2^). Asterisks indicate statistical signiﬁcance (*p*^6^ = 0.0003; *p*^7^ < 0.0001; Wilcoxon signed-rank test). **(G)** Ca^2+^ signal count in pTPCs (ctr: mean ± SD = 11.9 ± 9.2, median = 9; GSK: 3.3 ± 3.4, median = 2) or pSCs (ctr: mean ± SD = 5.5 ± 5.8, median = 4; GSK: mean ± SD = 2.8 ± 3.0, median = 2) per 10 min recordings. Asterisks indicate statistical signiﬁcance (*p*^8^ and *p*^9^ < 0.0001; Mann–Whitney *U* test). **(H)** Schematic depicting electroporation-based plasmid transfer to cells of the mouse seminiferous tubule. Co-expression of a fluorophore (mOrange2) allows identification of transfected cells in acute slices. **(I)** Original traces (Δ*F*/*F*_0_; Ca^2+^ concentration *vs.* time) recorded under control conditions (ctr 1; sham injection), scrambled RNA control (ctr 2), 5ppase expression, or IP_3_ receptor type 1 knock-down (IP3R1KD). **(J)** Raster plots depicting individual Ca^2+^ signals (or lack thereof) over time (5 min). **(K)** Quantification of residual cellular activity after gene transfer, derived from recordings as shown in (I). Asterisks indicate statistical signiﬁcance (*p*^10^ and *p*^11^ < 0.0001; *p*^12^ = 0.015; *p*^13^ = 0.0018; Fisher´s exact test). **(L)** Dot and violin plots quantify signal count in controls and cells that retain activity after IP3R1KD or 5ppase expression. Event numbers per 5 min are plotted for ctr 1 (mean ± SD = 22.6 ± 15, median = 21), ctr 2 (19.6 ± 12.6, median = 17), 5ppase (mean ± SD = 7.1 ± 8.3, median = 4), and IP3R1KD (7.9 ± 10.2, median = 4). Asterisks indicate statistical signiﬁcance (*p*^14^ and *p*^15^ < 0.0001; *p*^16^ = 0.001; *p*^17^ = 0.0014; Kruskal–Wallis with *post-hoc* Dunn test and Benjamini-Hochberg correction). Black dots represent mean, black diamonds display median values. The underlying numerical data for this figure is detailed in S1 Data.

Next, we asked which signaling mechanism(s) underlie each type of spontaneous Ca^2+^ activity. We used a variety of experimental approaches, ranging from pharmacology to protein knock-down, to interfere with critical Ca^2+^ signaling pathways (Fig 2A). Prolonged exposure of previously active cells to reduced extracellular Ca^2+^ conditions strongly diminished spontaneous activity in the vast majority of cells, an effect that consolidated over time and proved fully reversible (Fig 2B and 2D; S1 Table). In addition, lasting Ca^2+^ depletion of the ER by inhibition of the sarco/endoplasmic reticulum Ca^2+^-ATPase (SERCA) with either of two selective agents (thapsigargin or cyclopiazonic acid (CPA)) abolished cellular activity (Fig 2C and 2D). Notably, the plant extract thapsigargin and the mycotoxin CPA target different SERCA structures [41]. These findings indicate that, while extracellular Ca^2+^ influx is involved, Ca^2+^ release from the ER is essential for spontaneous activity within the seminiferous tubule.

Normally, store-operated Ca^2+^ entry (SOCE [42]) via STIM and ORAI proteins fuels SERCA-dependent restoration of ER Ca^2+^ levels [43]. We therefore asked whether block of store-operated ORAI channels also affects spontaneous Ca^2+^ activity. Compared to control conditions, exposure to the selective and potent ORAI inhibitor GSK-7975A (10 µM; 10 min [44]) substantially reduced the number of active cells per tubule area (Fig 2E and 2F). This effect became evident for both putative TPCs and Sertoli cells, as categorized by their distinct shape and location (Fig 2E). Moreover, cells that maintained residual activity in presence of the drug displayed fewer Ca^2+^ transients (Fig 2E and 2G). This decline in signal frequency appears more pronounced in putative TPCs, which under control conditions also show higher average event rates than putative Sertoli cells. Together, these results suggest that ORAI-mediated SOCE is required to maintain spontaneous Ca^2+^ signaling integrity, likely acting in a use-dependent manner.

To address the mechanistic basis of ER Ca^2+^ release we targeted inositol 1,4,5-trisphosphate (IP_3_) dependent signaling processes. After gene transfer into the mouse testis, both knock-down of IP_3_ receptor type 1 (IP3R1) and expression of 5-phosphatase (5ppase), an enzyme that hydrolyzes IP_3_ [45], strongly reduced spontaneous activity (Fig 2H–2L). While normalized numbers of active cells were only slightly, though significantly reduced (Fig 2I and 2K), activity rates dropped dramatically upon IP3R1 knock-down or 5ppase expression (Fig 2J and 2L). Together, our data suggest that, at least to a large extent, spontaneous Ca^2+^ signals in the mouse seminiferous tubule are mediated by IP_3_-dependent Ca^2+^ release from endoplasmic stores.

### Cell type-specific phenotyping reveals distinct patterns of Ca^2+^ activity in TPCs, spermatogonia, and Sertoli cells

**Fig 3:**
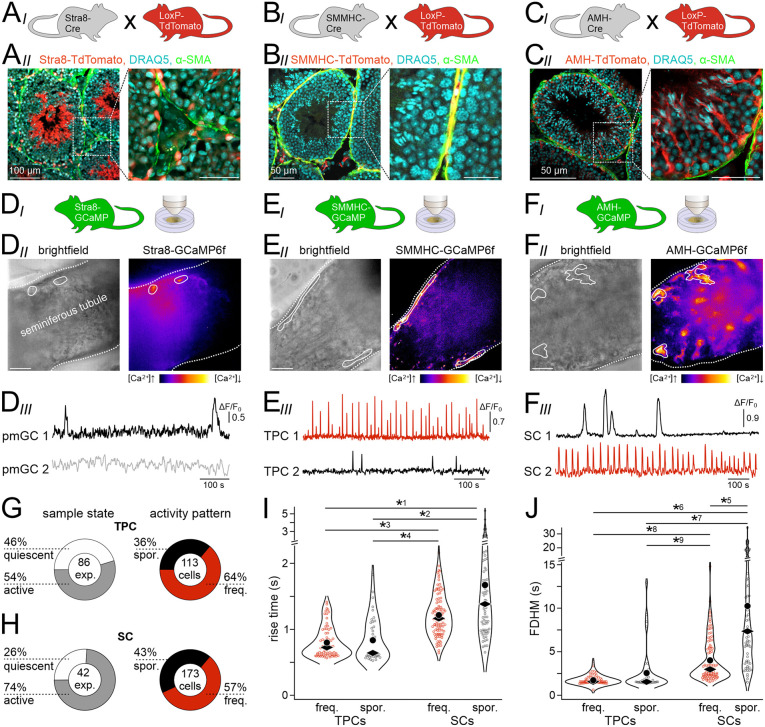
Cell type-specific phenotyping reveals distinct patterns of Ca^2+^ activity in TPCs, spermatogonia, and Sertoli cells. **(A–C)** The mouse Cre/Lox system enables conditional reporter protein expression in defined testicular cell types. Breeding schemes (**A**_***I***_–**C**_***I***_) and histology (**A**_***II***_–**C**_***II***_) demonstrate tdTomato expression in premeiotic germ cells **(A)**, TPCs **(B)**, and Sertoli cells **(C)**, driven by Stra8-Cre, SMMHC-CreER^T2^, and AMH-Cre mice, respectively. Confocal fluorescence images (A_*II*_–C_*II*_) and zoom-ins (white dashed rectangle) depict tdTomato expression (red), DRAQ5 nuclear staining (cyan), and immunochemical α-smooth muscle actin labeling of TPCs and vasculature (α-SMA; green). Scale bars in zoom-ins: 50 µm (A_*II*_) and 25 µm (B_*II*_ and C_*II*_). **(D–F)** Driving GCaMP6f expression, according to the above breeding strategy, in spermatogonia **(D)**, TPCs **(E)**, and Sertoli cells (**F**) allows cell type-specific fluorescence imaging of Ca^2+^ signals. Representative brightfield (left) and corresponding semi-confocal fluorescence micrographs (right; **D**_***II***_–**F**_***II***_) outline individual seminiferous tubules (dotted lines) and ROIs (white lines) that display spontaneous Ca^2+^ transients. Scale bars: 25 µm. Pseudocolors (inferno color map) indicate relative Ca^2+^ concentration. Example traces (ΔF/F_0_ *vs.* time) for each cell type are shown in (**D**_***III***_–**F**_***III***_). **(G, H)** Wheel charts quantifying whether Ca^2+^ signals were observed in TPCs (**G**) or Sertoli cells (**H**) in a given seminiferous tubule (left), and whether these signals occurred either sporadically (black) or frequently (red). **(I, J)** Dot and violin plots comparing kinetic features (rise time **(I)**; duration **(J)**) of Ca^2+^ signals within and between cell types (TPCs *vs.* Sertoli cells) and signal characteristics (frequent (red) *vs.* sporadic (black)). Black dots represent mean, diamonds display median values for both signal rise time [TPCs: mean ± SD = 0.8 ± 0.2 s (red) *vs.* 0.8 ± 0.3 s (black), median = 0.7 s (red) *vs.* 0.6 s (black); SCs: mean ± SD = 1.2 ± 0.3 s (red) *vs.* 1.7 ± 0.9 s (black), median = 1.2 s (red) *vs.* 1.4 s (black)] and signal duration [TPCs: mean ± SD = 1.7 ± 0.5 s (red) *vs.* 2.5 ± 2.5 s (black), median = 1.5 s (red) *vs.* 1.5 s (black); SCs: mean ± SD = 4.0 ± 2.5 s (red) *vs.* 10.3 ± 7.5 s (black), median = 3.0 s (red) *vs. 7.3* s (black)]. Asterisks indicate statistical signiﬁcance (*p*^1^– *p*^9^ < 0.0001; Kruskal–Wallis with *post-hoc* Dunn test and Benjamini-Hochberg correction). FDHM, full duration at half maximum; pmGC, premeiotic germ cell; SC, Sertoli cell. The underlying numerical data for this figure is detailed in S1 Data.

We next aimed to unequivocally identify which cell types generate spontaneous Ca^2+^ activity within seminiferous tubules. Conditional gene targeting via the Cre/Lox system allows cell type-specific expression of reporter proteins in the mouse testis [46]. In addition to previously established genetic labeling of TPCs using SMMHC-CreER^T2^ mice [30] we here employ specific driver lines (AMH-Cre, Stra8-Cre) that allow targeted testicular expression in Sertoli cells or premeiotic germ cells, respectively. Crossing animals from each driver line with Ai14D reporter mice generates offspring in which either spermatogonia (Stra8-Cre × Ai14D), TPCs (SMMHC-CreER^T2^ × Ai14D), or Sertoli cells (AMH-Cre × Ai14D) are fluorescently labeled by tdTomato expression (Fig 3A–3C and S2–S4 Movies).

To analyze cell type-specific spontaneous activity, we next applied the above labeling approach to express a genetically encoded Ca^2+^ indicator (GCaMP6f) in either TPCs, Sertoli or germ cells (Fig 3D_*I*_–3F_*I*_). While spontaneous Ca^2+^ signals are observed in each cell type (Fig 3D_*II&III*_–3F_*II&III*_), activity in premeiotic germ cells is rare. By contrast, we recorded Ca^2+^ signals from both TPCs and Sertoli cells in the majority of tubules tested (Fig 3G and 3H; S1 Table). Notably, for TPCs, only Ca^2+^ signals not associated with seminiferous tubule contractions [30] were considered for analysis. While, according to our coarse initial categorization (Fig 1C–1E), both TPCs and Sertoli cells exhibit frequent as well as sporadic activity (Fig 3G and 3H), analysis of individual signal kinetics suggests that essentially all TPC signals are brief Ca^2+^ transients with fast rise times and short durations. By contrast, Sertoli cells display either frequently occurring short signals or sporadic, but prolonged Ca^2+^ transients (Fig 3I and 3J). Notably, in line with the notion of use-dependent SOCE exhaustion upon Ca^2+^ gradient reversal or ORAI inhibition (Fig 2B–2G), brief 3-min exposure to reduced extracellular Ca^2+^ strongly diminished the number of active TPCs, whereas Sertoli cells were yet to be affected (S1 Fig).

Together, our experiments in seminiferous tubule slices demonstrate (*i*) that the Cre/Lox genetic toolkit allows selective labeling of spermatogonia (see Discussion), TPCs, or Sertoli cells, respectively; (*ii*) that spontaneous Ca^2+^ signals only rarely occur in spermatogonia, whereas both TPCs and Sertoli cells display considerable Ca^2+^ activity; and (*iii*) that these two somatic cell types exhibit distinct Ca^2+^ signaling patterns with slower, but more sustained signals being largely restricted to Sertoli cells.

### *In vivo* measurements consolidate concepts obtained from *in vitro* recordings

**Fig 4:**
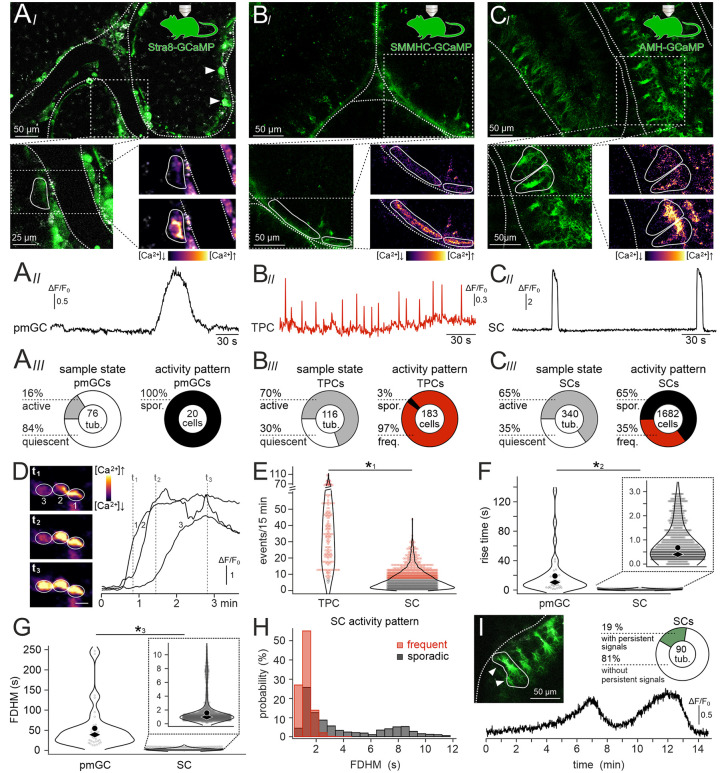
*In vivo* measurements consolidate concepts obtained from *in vitro* recordings. **(A–C)** Intravital multiphoton fluorescence microscopy of Ca^2+^ signals in spermatogonia **(A)**, TPCs **(B)**, and Sertoli cells **(C)**. GCaMP6f expression (green) is driven by Stra8-Cre, SMMHC-CreER^T2^, and AMH-Cre mice, respectively. Representative images (**A**_***I***_–**C**_***I***_) depict overviews (top; tubules outlined by dotted lines) and zoom-ins (bottom) corresponding to the regions outlined by white dashed rectangles. ROIs (white solid lines) label active cells at different time points. Pseudocolors (inferno color map) indicate relative Ca^2+^ concentration. (**A**_***II***_–**C**_***II***_) Example traces (GCaMP6f fluorescence intensity; Δ*F*/*F*_0_ *vs.* time) illustrate typical original recordings. (**A**_***III***_–**C**_***III***_) Wheel charts quantify whether Ca^2+^ signals were observed in a given seminiferous tubule (sample state; left), and whether activity occurred either sporadically (black) or frequently (red). **(D)** Wave-like spread of Ca^2+^ signals along chains of spermatogonia. Images (left) depict three adjacent cells (white ROIs; inferno color map) at three different time points (*t*_1_–*t*_3_). Corresponding traces (right) show fluorescence intensity over time. Scale bar: 10 µm. **(E)** Dot and violin plots comparing Ca^2+^ signal count over 15 min between TPCs and Sertoli cells. Colors indicate sporadic (≤7 events; gray) *vs.* frequent (red) activity. Asterisk indicates statistical significance (*p*^1^ < 0.0001; Mann–Whitney *U* test) **(F, G)** Dot and violin plots comparing kinetic features (rise time **(F)**; duration **(G)**) of individual Ca^2+^ signals between premeiotic germ cells (pmGCs) and Sertoli cells (SCs). Black dots represent means, black diamonds display median values for both 20%–80% rise time (pmGCs: mean ± SD = 19.1 ± 27.6 s, median = 10.4 s; SCs: mean ± SD = 0.7 ± 0.6 s, median = 0.4 s) and signal duration (pmGCs: mean ± SD = 54.7 ± 55.7, median = 38.3; SCs: mean ± SD = 1.6 ± 1.8, median = 1.0). Asterisks indicate statistical signiﬁcance (*p*^2^ and *p*^3^ < 0.0001; Mann–Whitney *U* test). **(H)** Probability distribution histogram for Sertoli cell Ca^2+^ signal durations, comparing frequent (red; *n* = 3,050) *vs.* sporadic (dark gray; *n* = 596) events. Note: while durations of frequent signals are normally distributed, sporadically occurring events show a bimodal distribution (Sarle’s bimodality coefficient: 0.71). **(I)** Sertoli cells show persistent Ca^2+^ elevations *in vivo*. Representative GCaMP6f fluorescence image (top, left) reveals increased Ca^2+^ levels in both basal and adluminal compartments (white arrow heads; dotted line indicates tubule wall). Wheel chart (top, right) quantifying the observations of persistent signals. Example trace (bottom; Δ*F*/*F*_0_ *vs.* time) illustrating the unique kinetics of persistent Ca^2+^ elevations. FDHM, full duration at half maximum; pmGC, premeiotic germ cell; SC, Sertoli cell. The underlying numerical data for this figure is detailed in S1 Data.

Using the above animal models for selective expression of GCaMP6f in premeiotic germ cells, TPCs, or Sertoli cells, respectively, we next asked whether apparently spontaneous Ca^2+^ signals also occur *in vivo*. To this end, we monitored cell type-specific activity by intravital multiphoton fluorescence microscopy in anesthetized animals [30]. Similar to observations in slices (Figs 1–3), Ca^2+^ signals in spermatogonia are rare events (Fig 4A). By contrast, in both TPCs and Sertoli cells activity is more prevalent (Fig 4B and 4C; S5 Movie; S1 Table). Notably, when spermatogonia show spontaneous activity, these Ca^2+^ signals always display slow onset kinetics, are prolonged, and lack any obvious regularity (Fig 4A_*II*_ and 4A_*III*_). Sometimes, when ‘chains’ of adjacent spermatogonia were visible within an optical section, we observed a wave-like spread of Ca^2+^ along these chains (Fig 4D), likely facilitated by intercellular bridges that result from incomplete germ cell divisions [47]. TPC *in vivo* activity, however, is exclusively characterized by frequent and fast Ca^2+^ transients (Fig 4B_*III*_). The majority of Sertoli cells, on the other hand, exhibit sporadic, slow activity (Fig 4C_*III*_).

The distinction between TPC and Sertoli cell signals also becomes apparent when quantifying the relative frequency of Ca^2+^ transients. While, on average, the vast majority of Sertoli cells displays less than one Ca^2+^ signal per minute, the frequency range among TPCs is much broader, with most cells generating between two and seven events per minute (Fig 4E). While TPC Ca^2+^ signals are generally too short-lived for detailed kinetics analysis (at our recording rate of 2 frames/s), signal comparison between spermatogonia and Sertoli cells demonstrates the more sluggish rise and longer duration of Ca^2+^ transients recorded from spermatogonia (Fig 4F and 4G). In Sertoli cells, fast and frequent signals are also clearly discernable from slower sporadic activity (Fig 4H). Notably, long-term recordings reveal an additional, so far unnoticed signal type in Sertoli cells. In about one-fifth of the tubules, some Sertoli cells exhibit persistent Ca^2+^ elevations that last for several minutes (Fig 4I and S6 Movie).

Together, our data show (*i*) that the signaling phenomena we observed in seminiferous tubule slices are largely recapitulated *in vivo*, with cell type-specific distinctions becoming even more apparent; (*ii*) that spermatogonia exclusively exhibit slow prolonged signals, while TPCs only show very fast Ca^2+^ transients, respectively; and (*iii*) that Sertoli cells can display a previously unnoticed, long-lasting Ca^2+^ signal that adds an additional dimension to the available signal space.

### Sertoli cell activity occurs in dynamic and stage-dependent hotspots

**Fig 5:**
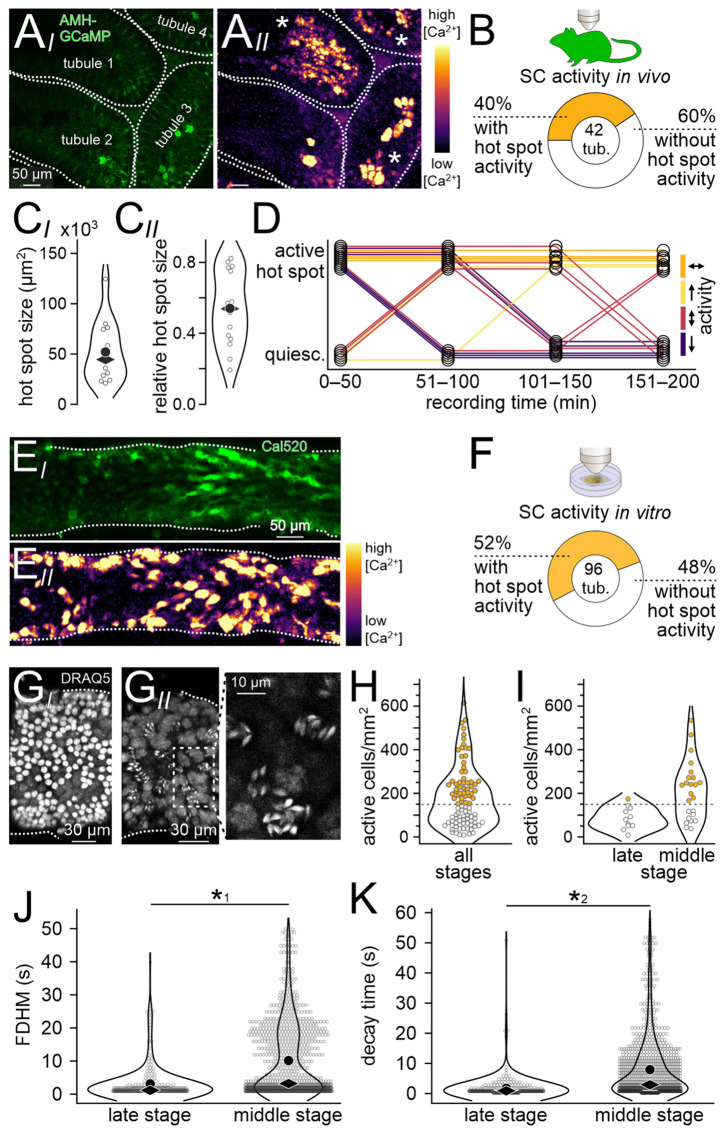
Sertoli cell activity occurs in dynamic and stage-dependent hotspots. *In vivo* multiphoton microscopy of GCaMP6f fluorescence in testes from AMH-Cre x Ai95D mice reveals Sertoli cell activity hotspots **(A–D)**. **(A)** Representative images depicting either a single-frame (**A**_***I***_) or the corresponding time-lapse maximum projection (**A**_***II***_) that identifies hotspots of activity (white asterisks). Individual tubules outlined by dotted white lines. Pseudocolors (inferno color map) indicate relative Ca^2+^ concentration. **(B)** Wheel chart quantifying *in vivo* observations of hotspot activity. **(C)** Dot and violin plots depicting absolute (left) and relative (right) hotspot size. Black dots represent mean, diamonds display median values (absolute area: mean ± SD = 52,603 ± 30,284 µm^2^, median = 45,118 µm^2^; relative size: mean ± SD = 0.54 ± 0.2, median = 0.54). **(D)** Long-term recurrent recordings from the same fields-of-view reveal dynamic switches between hotspot activity and quiescent states. Line colors indicate sustained (orange), gain (yellow), multiple switches (red), or loss (purple) of hotspot activity. **(E–K)** *In vitro* confocal fluorescence microscopy in Cal-520/AM-loaded isolated tubules reveals spermatogenic stage dependence of Sertoli cell activity hotspots. **(E)** Single-frame ((**E**_***I***_); green) and corresponding time-lapse maximum projection ((**E**_***II***_); inferno color map) confocal images depict hotspot activity. **(F)** Wheel chart quantifying *in vitro* observations. **(G)** Representative fluorescence images of tubule nuclei (DRAQ5; grayscale; z-stack intensity projections) in late (**G**_***I***_) *vs.* middle (**G**_***II***_) stage. Dashed rectangle in (G_*II*_) is magnified and shown as a single optical section through the apical epithelium. Note the clusters of highly condensed late spermatid nuclei. (**H**, **I**) Dot and violin plots depicting active cells per area from all measurements (**H**) and comparing those categorized as either late (left) or middle (right) stage **(I)**. Dotted horizontal line indicates the threshold (150 active cells/mm^2^) that distinguishes tubule areas designated as hotspots (yellow dots) from those less active (open circles). **(J, K)** Dot and violin plots comparing kinetic features (FDHM **(J)**; 80%–20% decay time **(K)**) of individual Ca^2+^ signals recorded from late *vs.* middle stage tubules. Black dots represent means, black diamonds display median values for both FDHM (late: mean ± SD = 2.9 ± 5.0 s, median = 1 s; middle: mean ± SD = 10.0 ± 11.3 s, median = 3 s) and decay time (late: mean ± SD = 1.8 ± 3.8 s, median = 1 s; middle: mean ± SD = 8.0 ± 10.5 s, median = 3 s). Asterisks indicate statistical signiﬁcance (*p*^1^ and *p*^2^ < 0.0001; Mann–Whitney *U* test). The underlying numerical data for this figure is detailed in S1 Data.

During *in vivo* recordings from Sertoli cells, we noticed that Ca^2+^ signals often occurred in tubule areas that contain clusters of active cells. We designated these active Sertoli cell assemblies as ‘hotspots’ (Fig 5A). Quantified over all recordings, ~ 40% of tubules display hotspot activity (Fig 5B; S1 Table). Yet, both absolute and relative (i.e., normalized to recorded tubule area) hotspot sizes are heterogeneous (Fig 5C), arguing against a spatially stereotypic activity pattern. Moreover, hotspot activity appears to be a transient phenomenon, since long-term *in vivo* observations reveal that seminiferous tubule regions can switch between hotspot activity and quiescent states (and *vice versa*; Fig 5D). Notably, both activity hotspots and their dynamic occurrence are also observed in juvenile mice *in vivo* (S2A and S2C–S2E Fig).

We hypothesized that hotspot activity could be related to junction disassembly and remodeling as observed within tubulobulbar complexes (TBCs) [39,48] that form in preparation of sperm release during middle stages (VI‒VIII; Fig 1A) of the spermatogenic cycle. The combination of live-cell imaging and *post-hoc* nuclear staining in slices allowed measurement of activity hotspots (Fig 5E and 5F) and subsequent identification of middle and late (IX‒XII) spermatogenic stages [3] according to their characteristic anatomical distribution of nuclei (Fig 5G [48]). We defined hotspot activity in slices according to a multimodal distribution of active Sertoli cells per area (Fig 5H), with regions containing >150 active cells/mm^2^ being considered a hotspot. Strikingly, when comparing activity in those tubules identified as either middle or late stage, hotspots are essentially confined to middle stages prior to spermiation (Fig 5I). Moreover, in middle stage tubules, Ca^2+^ signals display slower decay and, thus, they last significantly longer than the more transient signals observed during late stages (Fig 5J and 5K).

In summary, our experiments reveal that Sertoli cell activity is often clustered in hotspots, with changing activity states over time. Moreover, hotspot activity correlates with spermatogenic cycle stage, supporting the notion that local ER-mediated Ca^2+^ fluctuations in Sertoli cells may exert mechanistic control over events related to TBC formation and spermiation [39].

### Sertoli cell activity displays unique spatial features

**Fig 6:**
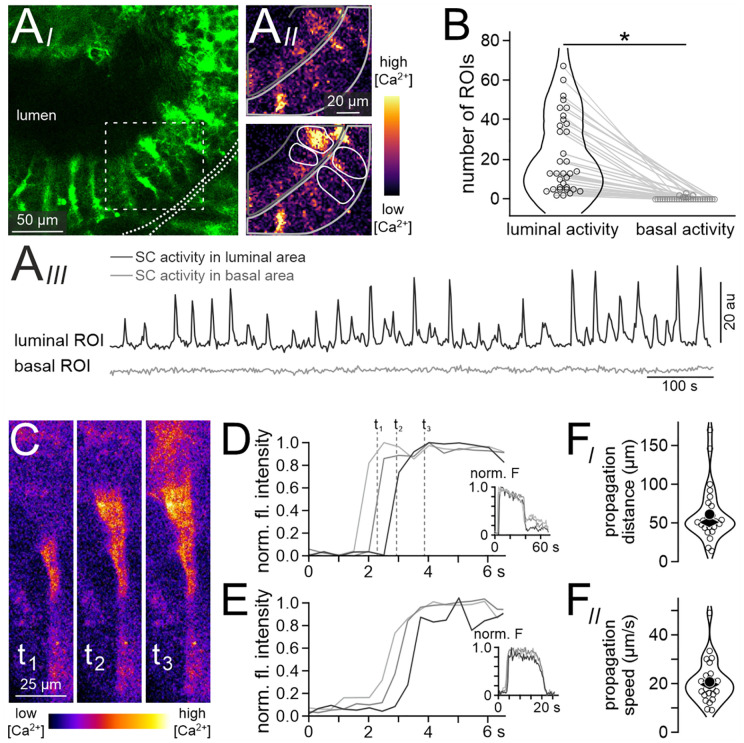
Sertoli cell activity displays unique spatial features. Intravital multiphoton imaging of GCaMP6f fluorescence in testes from AMH-Cre x Ai95D mice reveals that transient Sertoli cell signals are confined to the adluminal compartment **(A)**. Representative images (**A**_***I***_, **A**_***II***_) and corresponding original traces ((**A**_***III***_); fluorescence intensity *vs.* time). Overview maximum projection (A_*I*_) and zoom-in (white dashed rectangle) pseudocolor images at two different time points (A_*II*_; inferno color map; scale bar: 20 µm) reveal spatially limited activity. Separating ROIs as either adluminal or basal (gray areas in (A_***II***_)) identifies frequent Ca^2+^ transients in adluminal regions, whereas basal compartments remain quiescent (A_***III***_). **(B)** Paired dot plot comparing activity in adluminal *vs.* basal areas. Asterisk indicates statistical signiﬁcance (*p* < 0.0001; two-tailed Wilcoxon signed-rank test). **(C&D)** Pseudocolor images ((**C**); inferno map) at three different time points (*t*_1_–*t*_3_) and three corresponding traces from adjacent ROIs (**D**) illustrate an intracellular Sertoli cell Ca^2+^ wave recorded in acute slices. Inset: plotting the same three signals over a prolonged period demonstrates the transient signal character. **(E)** Example traces depicting a similar wave-like Ca^2+^ spread *in vivo*. **(F)** Dot and violin plots quantifying Sertoli cell Ca^2+^ wave propagation distance (**F**_***I***_) and speed **(F**_***II***_). Black dots represent means, diamonds display median values (distance: mean ± SD = 61.5 ± 34.4 µm, median = 51 µm; speed: mean ± SD = 20.6 ± 8.7 µm/s, median = 19.5 µm/s). au, arbitrary units. The underlying numerical data for this figure is detailed in S1 Data.

Another striking feature of spontaneous Sertoli cell activity is a fundamental difference between the cells’ adluminal and basal regions. With the notable exception of persistent Ca^2+^ elevations (Fig 4I), less long-lived Ca^2+^ transients are exclusively confined to adluminal Sertoli cell compartments (Fig 6A and 6B; S1 Table). In juvenile males, we recorded a similar pattern of spatial segregation (S2B Fig). This unexpected subcellular division into spatially segregated biochemical compartments is likely to exert distinct effects on the epithelial regions separated by the BTB.

Notably, in both *in vitro* and *in vivo* recordings, transient Sertoli cell Ca^2+^ signals frequently occurred as intracellular waves (Fig 6C–6E) that propagate with substantial speed over long distances (Fig 6F and S7 Movie). Together, while persistent Ca^2+^ elevations affect the entire Sertoli cell, more transient signals are restricted to adluminal cellular compartments and frequently display a wave-like character.

### Transient Ca^2+^ signals lack orchestration, whereas persistent activity is synchronized within and across tubules

**Fig 7:**
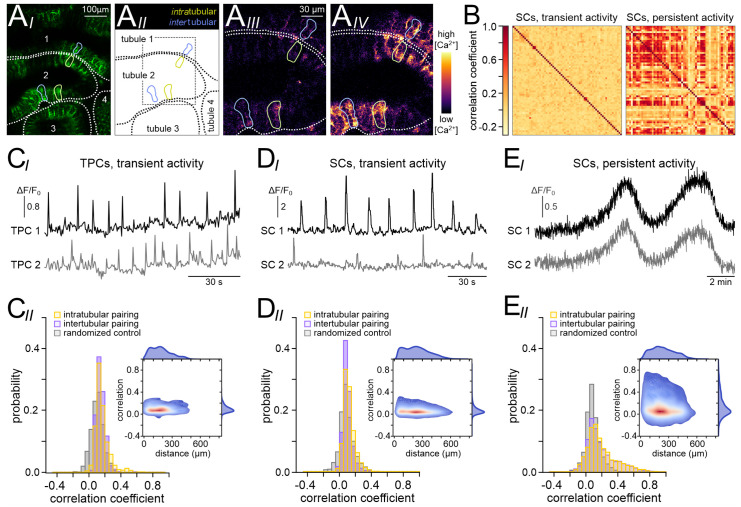
Transient Ca^2+^ signals lack orchestration, whereas persistent activity is synchronized within and across tubules. **(A)** Designation of *intra*tubular (pale green ROIs) *vs. inter*tubular (blue ROIs) pairs of active Sertoli cells. Overview multiphoton fluorescence image (**A**_***I***_) and corresponding tubule map **(A**_***II***_). Zoom-ins (**A**_***III***_, **A**_***IV***_), corresponding to dashed rectangle in **(A**_***II***_), depict GCaMP6f signals at two different time points. Pseudocolors (inferno color map) indicate relative Ca^2+^ concentration. **(B)** Pairwise correlation matrices for Sertoli cell Ca^2+^ signals categorized as either transient (left) or persistent (right). Each matrix corresponds to a representative experiment/field-of-view. Correlation coefficients shown as heat maps (fire color map). **(C–E)** Example trace pairs (GCaMP6f fluorescence intensity; Δ*F*/*F*_0_ *vs.* time; (**C**_***I***_–**E**_***I***_)) and correlation probability histograms (**C**_***II***_–**E**_***II***_) are shown for TPC transient activity (**C**) as well as both transient (**D**) and persistent (**E**) activity in Sertoli cells. Individual histograms compare control pairs randomly picked from different recordings (light gray; *n* = 625 (C_***II***_), 1,975 (D_*II*_ and E_*II*_)) with all *intra*tubular (yellow; *n* = 545 (C_***II***_), 213,384 **(D**_***II***_), 10,432 **(E**_***II***_)) and *inter*tubular (violet; *n* = 1,217 (C_***II***_), 107,548 **(D**_***II***_), 10,898 **(E**_***II***_)) signal pairs recorded simultaneously. Insets show kernel density estimate plots that, for each cell pair, relate correlation coefficients and the distance between ROI centroids. The underlying numerical data for this figure is detailed in S1 Data.

In each field-of-view, we usually record from several seminiferous tubules simultaneously (Fig 7A_*I*_ and S1 Table). This configuration allows us to investigate whether activity patterns are correlated between individual cells—both within the same tubule (i.e., *intra*tubular) and between different tubules (i.e., *inter*tubular; Fig 7A_*II–IV*_). Pairwise correlation analysis of all active cells in a given field-of-view (Fig 7B) revealed strong differences in signaling synchrony depending on activity pattern. In fact, while transient activity in both TPCs and Sertoli cells appears mostly devoid of correlation (Fig 7C and 7D), persistent Sertoli cell activity often shows a high degree of synchrony. This is evident not only for intratubular, but also for intertubular cell pairs (Fig 7E and S6 Movie). Notably, synchronous activity appears somewhat dependent on cell pair distance, indicating that local environmental conditions, rather than tubular boundaries, control synchrony in sustained Sertoli cell activity (Fig 7E_*II*_, inset).

### Gonadotropins affect Sertoli cell signaling *in vivo*

**Fig 8:**
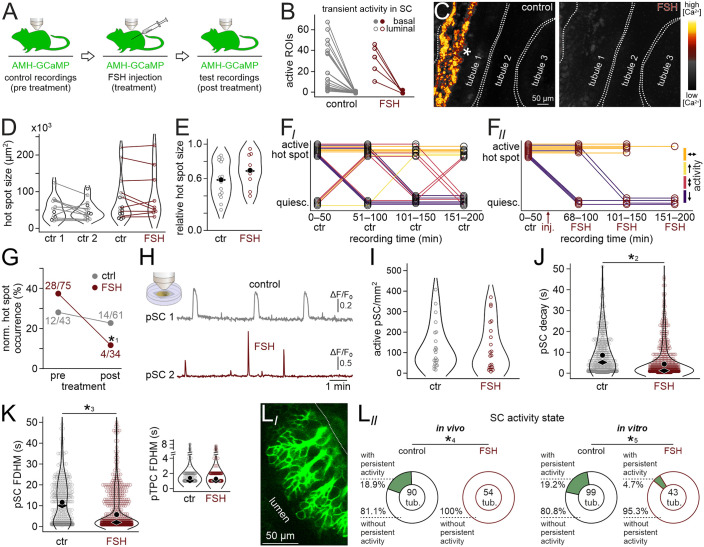
Gonadotropins affect Sertoli cell signaling *in vivo.* **(A)** Schematic illustrating the experimental strategy. **(B)** Paired dot plots comparing transient activity in adluminal *vs.* basal Sertoli cell areas under control conditions (gray) as well as after systemic injection of FSH (red). **(C)** Example maximum projections of GCaMP6f fluorescence in the same field-of-view before (left) and after (right) FSH treatment. Pseudocolors (fire color map) indicate relative Ca^2+^ concentration. Asterisk indicates hotspot activity. **(D, E)** Dot and violin plots depicting absolute (**D**) and relative (**E**) hotspot size. Color code as in (B). Absolute values (D) are compared before *vs.* after treatment (lines label paired regions). Black dots in (E) represent means, diamonds display median values (mean ± SD = 0.54 ± 0.2 (control), 0.65 ± 0.19 (FSH); median = 0.54 (control), 0.64 (FSH)). **(F)** Long-term recurrent recordings from the same fields-of-view under control conditions ((**F**_***I***_); see also Fig 5D) as well as before *vs.* after FSH (**F**_***II***_) treatment. Line colors indicate sustained (orange), gain (yellow), multiple switches (red), or loss (purple) of hotspot activity. Note that data is only included if a tubule displayed hotspot activity at least once and if at least one pre- and one post-treatment period had been measured per field-of-view. **(G)** Percentage of hotspot occurrence during pre- *vs.* post-treatment periods. Note that all tubules are included, i.e., independent of the inclusion criteria described in (F). Asterisk indicates statistical signiﬁcance (*p*^1^ = 0.007; Fisher’s Exact test). **(H–K)** Analysis of *in vitro* experiments. **(H)** Original 10 min recordings from putative Sertoli cells show Ca^2+^ concentration (Δ*F*/*F*_0_) over time under control conditions (top) or during FSH treatment (bottom; 33 ng/ml; 20 min preincubation). **(I–K)** Dot and violin plots depicting active cells per area (**I**) as well as signal kinetics (signal decay **(J)**; FDHM **(K)**). Inset in (K) shows a corresponding analysis of data obtained from putative TPCs. Black dots in (J&K) represent means, black diamonds display median values for both decay time (ctr: mean ± SD = 8.6 ± 9.1 s, median = 5 s; FSH: mean ± SD = 4.2 ± 7.0 s, median = 1 s) and FDHM (ctr: mean ± SD = 11.6 ± 10.5 s, median = 10 s; FSH: mean ± SD = 5.8 ± 8.7 s, median = 2 s). Asterisks indicate statistical signiﬁcance (*p*^2^ and *p*^3^ < 0.0001; Mann–Whitney *U* test). **(L)** Gonadotropins reduce persistent Sertoli cell activity. Multiphoton micrograph (**L**_***I***_) depicting a lasting increase in GCaMP6f fluorescence within neighboring Sertoli cells. Wheel charts (**L**_***II***_) quantifying the *in vivo* (left) and *in vitro* (right) occurrence of persistent Ca^2+^ elevations under control conditions *vs.* FSH treatment, respectively. Asterisks indicate statistical signiﬁcance (*p*^4^ = 0.0003; *p*^5^ = 0.02; Fishers Exact test). The underlying numerical data for this figure is detailed in S1 Data.

Endocrine control of spermatogenesis functionally converges on Sertoli cells [10]. Therefore, we next asked whether and, if so, to what extent experimentally evoked gonadotropin surges affect Sertoli cell signaling *in vivo*. We monitored Sertoli cell Ca^2+^ signals both prior to and after systemic injection of either FSH or LH (Figs 8A and S3A; S1 Table) at supraphysiological concentrations. We then compared the occurrence and phenotypes of different Ca^2+^ signaling patterns pre- *versus* post-treatment between control conditions (sham), FSH or LH injections. Under each condition, transient Sertoli cell Ca^2+^ signals remain confined to the adluminal cellular compartments (Figs 8B and S3B). With respect to Ca^2+^ signaling hotspots, we also fail to observe any treatment-dependent differences in either absolute or relative hotspot size (Figs 8C–8E, S3C, and S3D). The same holds true for *in vivo* activity upon FSH exposure in juvenile males (S4A–S4C Fig). Notably, however, the transient, dynamic nature of hotspot occurrence changes dramatically as a result of gonadotropin surges. While, under control conditions, seminiferous tubule regions frequently alternate between broadly active and quiescent states in both adult and juvenile male mice (Figs 5D, 8F_*I*_ and S2E), systemic FSH treatment in particular silences previously active areas (Figs 8F_*II*_, S3E, and S4D). This effect is also evident from normalized quantification of general hotspot occurrence (Figs 8G, S3F, and S4E). Together, our findings suggest that gonadotropins, and FSH in particular, have a dampening effect on widespread Sertoli cell *in vivo* Ca^2+^ activity.

To explore whether FSH directly affects Sertoli cell Ca^2+^ signaling at quasi-physiological concentrations, we challenged isolated seminiferous tubules with FSH (33 ng/ml) for 30 min and monitored spontaneous Ca^2+^ mobilization in putative Sertoli cells. As expected from the time course of inhibition observed *in vivo* (Fig 8F_*II*_) 30 min gonadotropin treatment had essentially no effect on the count of active cells per area (Fig 8H and 8I). However, FSH exposure clearly affected Sertoli cell Ca^2+^ signal kinetics. In the presence of FSH, Ca^2+^ transients displayed substantially faster decay, leading to much shorter individual events, an effect not observed in putative TPCs (Fig 8J and 8K).

Finally, gonadotropins dramatically reduced (LH) or even abolished (FSH) persistent Sertoli cell activity (Figs 8L and S3G). While we recorded persistent Ca^2+^ elevations *in vivo* among 19% of all tubules under control conditions (Figs 4I and 8L_*II*_; S6 Movie), Sertoli cells exhibited persistent activity in only 9.8% of tubules during LH treatment (S3G Fig). Such persistent signals proved entirely absent upon FSH treatment (Fig 8L_*II*_). A similar inhibitory effect emerged *in vitro*. Within just 30 min of FSH exposure, the fraction of isolated tubules that display persistent Ca^2+^ elevations declined from 19% to 5% (Fig 8L_*II*_). Notably, *in vivo* recordings from juvenile male mice revealed no effect of FSH treatment on persistent signals, suggesting age-dependent FSH susceptibility (S4F Fig).

Together, our results demonstrate that gonadotropins, and especially FSH, directly affect Sertoli cell Ca^2+^ signaling both *in vitro* and *in vivo*, with effects on persistent activity proving age-dependent. While more transient, scattered, and strictly adluminal activity appears essentially unaffected, both hormones exert profound effects long-term, modulating signal kinetics and dampening both hotspot and persistent Ca^2+^ activity.

## Discussion

The physiological principles that control mammalian spermatogenesis still largely remain a biological black box. Here, we monitor cytosolic Ca^2+^, a universal tool of signal transduction, within the three cell types that constitute the mouse seminiferous tubule, i.e., TPCs, Sertoli and germ cells. Both *in vitro* and *in vivo* experiments reveal distinct, cell-type-specific signal patterns. These (*i*) display unique spatiotemporal fingerprints, (*ii*) rely on IP_3_-dependent Ca^2+^ release from endoplasmic stores, and (*iii*) are modulated by gonadotropins.

Ca^2+^, the most tightly regulated ion within membrane-bound organisms, affects the function of thousands of proteins [14]. Accordingly, Ca^2+^ signals exert pleiotropic actions, serving as both positive and negative switches in a plethora of cellular processes [13]. Its exact physiological effects are determined by the unique spatiotemporal profile of any given Ca^2+^ signal. In intact seminiferous epithelia, physiological Ca^2+^ signals have only been measured in TPCs [30]. Our studies in both acute seminiferous sections and in intact testes demonstrate a rich portfolio of cell type-dependent activity phenotypes. While spermatogonia rarely display Ca^2+^ transients, such sparse events, when occurring, are prolonged, show slow kinetics, and lack obvious regularity. Note that our findings do not exclude any role(s) of spermatogonial Ca^2+^ signals that act on time scales beyond our measurement window and/or temporal resolution. Profound but slow physiological events, such as mitotic entry or commitment to meiosis, might be characterized by unique Ca^2+^ signatures that evade our present imaging protocol.

TPCs and Sertoli cells display considerable Ca^2+^ activity. In TPCs, fast signals occur at relatively high frequencies and show some degree of periodicity. Sertoli cell signals, on the other hand, range from short-lived to longer to even sustained Ca^2+^ transients. Notably, Sertoli cell Ca^2+^ signals display several distinctive features. As architectural units of the seminiferous epithelium these columnar cells show transient signals that are restricted to adluminal compartments and sometimes propagate as waves. By contrast, sustained Ca^2+^ elevations affect the entire Sertoli cell. Frequently, Sertoli cell activity is clustered. These hotspots are heterogeneous in size and temporally dynamic. When Sertoli cell activity persists for prolonged periods, signals are often synchronized—a phenomenon that extends across individual tubules.

In Sertoli cells, the ER forms a network throughout the entire cell with cisternae acting as consistent features of ectoplasmic specializations [40], one of the defining features of morphologically mature mammalian Sertoli cells [39,48]. Combining features of adherens and tight junctions, ectoplasmic specializations facilitate the extensive junction-restructuring events in the seminiferous epithelium [49], indicating important roles for Ca^2+^ during junction remodeling [40]. Notably, TBCs form only in regions previously occupied by ectoplasmic specializations [39,48]. Here, our mechanistic analysis indicates that Ca^2+^ release from the ER is essential for spontaneous activity within the seminiferous tubule. This effect is, at least in part, IP_3_-dependent. While Ca^2+^ from the extracellular space is also required, its influx could, ultimately, be necessary to replenish storage organelles [13]. The immediate impact of Ca^2+^ gradient reversal on TPC signaling (S1 Fig) indicates a dependence on extracellular Ca^2+^ influx on a per-event basis, whereas Sertoli cells can maintain Ca^2+^ signaling on ER-based mechanisms for longer periods.

IP_3_-triggered Ca^2+^ mobilization from ER stores involves IP3R activation, Ca^2+^ release, sensation of ER depletion by stromal interaction molecule 1 (STIM1) and, ultimately, activation of store-operated ORAI channels in the plasma membrane [43]. This SOCE pathway [42] allows the SERCA pump to restore ER Ca^2+^ levels. In Sertoli cells, the molecular machinery associated with ER-generated Ca^2+^ fluxes—including IP3R1, SERCA2, STIM1, and ORAI1—is present in structures directly related to junction remodeling and spermiation such as TBCs [39,48]. While knockout of IP3R2 or IP3R3 alone does not affect spermatogenesis [50], IP3R1 knockdown has been shown to alter Sertoli cell actin dynamics, preventing maturation of TBC bulbs [48]. Homozygous knockout of SERCA2, the major ER Ca^2+^-ATPase in non-muscle tissues [51], causes embryolethality [52]. While mice lacking expression of both STIM1 and ORAI1 die early postnatally [53], mice solely deficient for ORAI1 survive, though male *Orai1*^−/−^ mice are sterile and display prominent defects in late-stage elongated spermatid development [54]. Results from both our pharmacological studies and genetic manipulations are in line with the notion that seminiferous tubule Ca^2+^ signaling is largely driven by mechanisms controlling Ca^2+^ release from the ER. Extending previous work [48], we propose that IP3R1, SERCA2, and ORAI1 orchestrate these processes, particularly at Sertoli cell contact sites between ER cisternae and the plasma membrane.

Support for the model outlined above stems from our finding that hotspot activity appears generally confined to stages of the spermatogenic cycle prior to / during spermiation, which are characterized by adluminal clusters of late spermatids and surrounding hook-like apical TBC processes of Sertoli cells. Once late spermatids are absent from the epithelium (late stages IX–XII) hotspot activity essentially ceases. The fact that those transient signals underlying activity hotspots are strictly restricted to adluminal Sertoli cell domains further confirms our hypothesis.

Our own previous results support a concept of Ca^2+^ signaling cross-talk between the ER and mitochondria in Sertoli cells [33]. Mitochondrial Ca^2+^ uptake and release shape the spatiotemporal ‘fingerprint’ of Sertoli cell Ca^2+^ signals *in vitro*. Here, mitochondrial Ca^2+^ accumulation initially occurs in distal areas, whereas Ca^2+^ buffering in perinuclear regions is detected with some delay. This could reflect a privileged localization of some mitochondria in proximity to local Ca^2+^ hotspots [55].

Ca^2+^ signals encode information within and between cells. The relevant signal parameters that encode information include kinetics, amplitude, spread, and frequency [13]. In fact, a frequency code might offer enhanced signaling fidelity [16]. Both cytosolic Ca^2+^ oscillations and saltatory Ca^2+^ waves typically depend on IP_3_ receptor activation, resulting Ca^2+^ release, and feedback of Ca^2+^ itself on the receptors [13]. The fire-diffuse-fire scheme underlying wave-like Ca^2+^ signal propagation [29] has also been observed in several other cell types [16]. We observe oscillatory Ca^2+^ signals in both Sertoli cells and, more pronounced, in TPCs. As we here focus exclusively on TPC Ca^2+^ elevations that occur in absence of coordinated seminiferous tubule contractions [30], it is tempting to speculate that these non-synchronous signals serve to sustain spontaneous low-amplitude ‘vibratory’ rippling movements [56] that likely maintain a constant smooth muscle tone in the tubular wall.

Sertoli cell Ca^2+^ signals are much more diverse, ranging from brief adluminal transients to regenerative saltatory waves to persistent Ca^2+^ elevations that spread throughout the entire cytosol. This complexity allows for highly dynamic signaling and, as such, meets the versatile functional demands faced by Sertoli cells during the spermatogenic cycle [57]. Stem cell niche formation, dynamic BTB restructuring while maintaining immune privilege, and coordination of synchronized stage transitions require a tailored repertoire of Ca^2+^ signals. Our findings indicate that the BTB also segregates the Sertoli cell cytosol into two biochemically isolated compartments. As argued above, adluminal Ca^2+^ transients might promote the disassembly of ectoplasmic specializations during spermiation [40]. By contrast, long-lasting cell-wide Ca^2+^ signals likely recruit transcription factors and, thus, exert long-term changes in gene expression [25] required for epithelial cyclicity.

Expressing both androgen and FSH receptors [11], Sertoli cells are the ultimate target of neuroendocrine activity along the hypothalamic–pituitary–testicular axis. When we experimentally evoked suprathreshold gonadotropin surges *in vivo*, we observed profound effects on Sertoli cell signaling. Both hotspot activity and persistent Ca^2+^ elevations were dramatically reduced or, upon FSH injections, even abolished. It is thus likely that the occurrence of widespread Sertoli cell Ca^2+^ activity coincides with the cycle stage-specific expression of FSH receptors [58]. *Vice versa*, transient and strictly adluminal Ca^2+^ signals could mark those epithelial stages characterized by low FSH receptor expression. The fact that we frequently observed synchrony in persistent Sertoli cell activity, which extends beyond tubular boundaries, supports the notion of endocrine control. In addition, FSH has been reported to regulate BTB dynamics by controlling the expression of junction proteins [12].

*In vitro* experiments allow us to monitor whether physiological serum concentrations of FSH directly affect seminiferous tubule Ca^2+^ signaling. In adult male C57BL/6 mice, serum FSH levels between 10 ng/ml and 75 ng/ml (33 ± 26 ng/ml; mean ± SD) have been reported [59–63]. We therefore challenged isolated tubules with 33 ng/ml FSH. While *in vitro* experiments allow precise control of hormone concentration, they lack the power of prolonged *in vivo* observations over several hours. Long-term effects, such as hotspot dynamics and their endocrine control, thus evade *in vitro* observation. Nonetheless, physiological FSH concentrations directly affect spontaneous Sertoli cell Ca^2+^ signal kinetics in isolated tubules, strongly reducing their duration and thus likely their impact.

When comparing Sertoli cell Ca^2+^ dynamics and their susceptibility to FSH in adult *versus* juvenile males, we note that patterns of transient and spatially restricted activity (i.e., hotspots and adluminal confinement) are very similar. By contrast, persistent and spatially uniform Ca^2+^ elevations, while occurring somewhat less frequently in juveniles, remain unaltered by FSH. While this intriguing discrepancy will have to be addressed in future studies, our observation substantiates that transient and persistent Ca^2+^ signals are controlled by different mechanisms, mediate different physiological processes, and are governed by different endocrine programs.

Cre/Lox conditional gene targeting enables cell type-specific expression of fluorescent Ca^2+^ reporters. In the testis, both the AMH-Cre and SMMHC-CreER^T2^ driver lines show exquisite selectivity for Sertoli cells and smooth muscle cells, respectively. In Stra8-Cre mice, however, we frequently noted ‘leaky’ reporter protein expression in somatic cells, sometimes even precluding reliable analysis of premeiotic germ cells. Nonetheless, their basal epithelial position and uniform spherical shape allowed unambiguous identification of spermatogonia in many experiments (see Materials and methods). However, we cannot rule out that such rather conservative categorization might have excluded some genuine germ cells from analysis. Fluorescence-activated cell sorting or any analysis of bulk Ca^2+^ levels that lacks anatomical information should, however, not be performed in offspring of Stra8-Cre mice. Another caveat is the use of anesthetized animals. While we do not expect any direct effects of anesthesia on testicular signaling, hypothalamic control of neuroendocrine pathways might be affected [64]. Third, while we carefully selected those transversal optical sections during *in vivo* recordings that allowed imaging of entire Sertoli cells, we cannot rule out that interstitial vasculature could sometimes have decreased fluorescence signal strength during measurements from basal Sertoli cell regions. Last, by acting as Ca^2+^ buffers, any indicator itself alters cellular Ca^2+^ signaling. Moreover, for very fast signals, as observed in TPCs, the imaging frame rates reached here limit or even preclude kinetics analysis. Faster imaging—at the expense of resolution and/or field-of-view size—and constantly improving GCaMP reporter proteins [65] will enable a more exact analysis of very short-lived elementary Ca^2+^ signals such as sparks or puffs [16] in the future.

Notably, a better understanding of the physiological Ca^2+^ signaling mechanisms that control seminiferous tubule function and spermatogenesis will prove essential to assess and comprehend aberrant Ca^2+^ signals under pathological conditions. Our findings provide a solid foundation for future studies aimed to identify signaling deficits that impair testicular function and, in extreme cases, might cause male infertility.

## Materials and methods

### Animals

Mice were maintained and sacrificed according to European Union legislation (Directive 2010/63/EU) and recommendations by the Federation of European Laboratory Animal Science Associations (FELASA). All experimental procedures were approved by the State Agency for Nature, Environment and Consumer Protection (LANUV; protocol number/ AZ 84-02.04.2016.A371). When possible, mice were housed in littermate groups of both sexes (room temperature (RT); 12:12 h light-dark cycle; food and water available *ad libitum*). For most experiments, we used adult (>12 weeks) males, which were sacrificed by cervical dislocation after sedation with 4% isoflurane. When investigating juvenile mice (S2 and S4 Figs), we used males at postnatal days 26–28.

We used C57BL/6J mice (Janvier Labs, France) as well as offspring from crossing either SMMHC-CreER^T2^ (JAX #019079) [66], B6.FVB-Tg(Stra8-icre)1Reb/LguJ (JAX#017490) [67], or 129S.FVB-Tg(Amh-cre)8815Reb/J (JAX #007915) [68] mice with either Ai95D (JAX #028865) [69] or Ai14D (JAX #007914) [70] mice, respectively.

### Chemicals and solutions

The following solutions were used:

(**S**_**1**_) 4-(2-Hydroxyethyl)piperazine-1-ethanesulfonic acid (HEPES) buffered extracellular solution containing (in mM) 145 NaCl, 5 KCl, 1 CaCl_2_, 0.5 MgCl_2_, 10 HEPES; pH = 7.3 (adjusted with NaOH); osmolarity = 300 mOsm (adjusted with glucose).

(**S**_**2**_) Oxygenated (95% O_2_, 5% CO_2_) extracellular solution containing (in mM) 120 NaCl, 25 NaHCO_3_, 5 KCl, 1 CaCl_2_, 0.5 MgCl_2_, 5 N,N-bis(2-hydroxyethyl)-2-aminoethanesulfonic acid (BES); pH = 7.3; 300 mOsm (adjusted with glucose).

(**S**_**3**_) Extracellular low Ca^2+^ solution containing (in mM) 145 NaCl, 5 KCl, 0.5 MgCl_2_, 10 HEPES; pH = 7.3 (NaOH); osmolarity = 300 mOsm (adjusted with glucose); [Ca^2+^]_free_ = 12 nM (1 mM EGTA, 0.1 mM CaCl_2_).

(**S**_**4**_) Oxygenated (95% O_2_, 5% CO_2_) extracellular low Ca^2+^ solution containing (in mM) 120 NaCl, 25 NaHCO_3_, 5 KCl, 0.5 MgCl_2_, 5 BES; pH = 7.3; 300 mOsm (adjusted with glucose); [Ca^2+^]_free_ = 12 nM (1 mM EGTA, 0.1 mM CaCl_2_).

Free Ca^2+^ concentrations in **S**_**3**_ and **S**_**4**_ were calculated using WEBMAXCLITE v1.15 (RRID:SCR_000459). If not stated otherwise, chemicals were purchased from Sigma (Schnelldorf, Germany). Thapsigargin was purchased from Tocris Bioscience (Bristol, UK). Cal-520/AM was purchased from Biomol (Hamburg, Germany). GSK-7975A was purchased from MedChemExpress (Sollentuna, Sweden). FSH was purchased from Abcam (Cambridge, UK). Final solvent concentrations were ≤0.1%.

### Stimulation

For focal stimulation, solutions and agents were applied from air pressure-driven reservoirs via an 8-in-1 multi-barrel ‘perfusion pencil’ (AutoMate Scientific; Berkeley, CA). Low [Ca^2+^]_e_ solutions (**S**_**3**_ and **S**_**4**_) as well as both thapsigargin and CPA were applied via both the bath (1 µM thapsigargin; 3 µM CPA) and perfusion pencil (10 µM thapsigargin; 30 µM CPA). GSK-7975A (10 µM) and FSH (33 ng/ml) were applied via bath exchange. To ensure depletion of Ca^2+^ stores by thapsigargin or CPA, we monitored intracellular Ca^2+^ levels during drug treatment (0.2 Hz frame rate). Upon administration, transient Ca^2+^ elevations lasted 30–40 min. After baseline Ca^2+^ levels were restored, slices were again imaged at 2 Hz frame rate. Control recordings, omitting thapsigargin and CPA, were performed under the same conditions.

### Slice preparation

Acute seminiferous tubule slices were prepared as previously described [30,71] with minor modifications. Briefly, seminiferous tubules were isolated after *tunica albuginea* removal, embedded in 4% low-gelling temperature agarose (VWR, Erlangen, Germany), and 250 µm slices were cut with a VT1000S vibratome (Leica Biosystems, Nussloch, Germany; RRID:SCR_016495). Acute slices were stored in a submerged, oxygenated storage container (**S**_**2**_; RT). When using testicular tissue from Ai95D mice, slices were protected from light during storage to avoid GCaMP6f bleaching.

### Systemic gonadotropin administration

During *in vivo* experiments, we injected a total volume of 50 µl i.p. (25 µl gonadotropin solution; 25 µl saline). To mimic a supraphysiological gonadotropin surge in an adult mouse (~30 g) with a total blood volume of ~2 ml, we injected 501 ng FSH and 90 ng LH, respectively, leading to calculated initial serum levels of 250.5 ng/ml (FSH) or 45 ng/ml (LH).

### Testis electroporation

Gene transfer into mouse testis followed previously published protocols [72,73]. Mice (P21–P25) were anesthetized by intraperitoneal injection of sodium pentobarbital (Kyoritsu Seiyaku Co., Tokyo, Japan). After plasmid DNA (5 µl; 1 µg/µl in H_2_O) injection into the testis via glass capillaries, electric pulses (40–50 V; 50 ms; 5x; 950 ms intervals) were delivered by an electroporator (CUY21, Nepa Gene, Chiba, Japan) using forceps-shaped electrodes (CUY650P3, Unique Medical Imada, Aichi, Japan). The pCAGGS plasmid vector was used for coexpression of the following construct combinations: 5PPase + mOrange2, miRNA against ITPR1 + mOrange2, miRNA (scrambled sequence) against ITPR1 + mOrange2. miRNA sequence for ITPR1 knock-down: 5′- TGCTGTTCATTTCCAGCCTCGTCCAAGTTTTGGCCACTGACTGACTTGGACGACTGGAAATGAA-3′

### Immunochemistry and tissue clearing

For immunochemistry of testicular cryosections, testes were fixed with 4% (w/v) paraformaldehyde (PFA) in PBS^−/−^ (10 mM, pH 7.4; ≥ 12 h; 4 °C) and subsequently cryoprotected in PBS^−/−^ containing 30% sucrose (≥24 h; 4 °C). Samples were then embedded in Tissue Freezing Medium (Leica Biosystems), sectioned at 20 µm on a Leica CM3050S cryostat (Leica Biosystems; RRID:SCR_020214), and mounted on Superfrost Plus slides (Menzel, Braunschweig, Germany). Next, sections were washed (3 × 5 min; PBS^−/−^). For blocking, sections were incubated in PBS^−/−^ containing Triton X-100 (0.3%), NaN_3_ (0.02%), normal goat serum (5%), and BSA (10 mg/ml) for 1 h (RT). After washing (PBS^−/−^ containing BSA (10 mg/ml); 2 × 5 min), sections were incubated with FITC-conjugated monoclonal anti-actin, α-smooth muscle (α-SMA-FITC, cat # F3777; Merck, Darmstadt, Germany) antibody (1:500 in PBS^−/−^ containing BSA (10 mg/ml); 16 h; 4 °C). Excess antibodies were removed by washing (PBS^−/−^ containing BSA (10 mg/ml); 2 × 5 min). For nuclear counterstaining, sections were then incubated in PBS^−/−^ containing DRAQ5 (1:1,000; 15 min; RT; Thermo Fisher Scientific, cat # 65-0880-96). Fluorescence images were taken using an inverted scanning confocal microscope (TCS SP8 DLS; Leica Microsystems) equipped with a 20 × 0.75 NA oil immersion objective (HC PL APO 20x/0.75 IMM CORR CS2, Leica Microsystems). To control for non-specific staining, experiments in which the primary antibody was omitted were performed in parallel with each procedure. Digital images were uniformly adjusted for brightness and contrast using LAS X software (Leica Microsystems).

For *post-hoc* spermatogenic cycle stage determination in isolated tubules, agarose-embedded sections were fixed (4% PFA in PBS^−/−^; ≥16 h) and nuclei were then stained with DRAQ5 (1:1,000; 30 min; RT; PBS^−/−^). Z-stacks of samples were imaged using a Leica TCS SP8 STED confocal microscope at 20× magnification (HC PL APO 20×/0.80 DRY; Leica Microsystems) and a pulsed tuneable white light laser (647 nm).

For testicular tissue clearing, we adopted the CLARITY method [74] with minor modifications [30,75]. Briefly, testes were fixed (>12 h; 4 °C) in hydrogel fixation solution containing 4% acrylamide, 0.05% bis-acrylamide, 0.25% VA-044 Initiator, 4% PFA in PBS^−/−^ to maintain structural integrity. After hydrogel polymerization (3 h; 37 °C), lipids were removed by incubation in 4% sodium dodecyl sulfate (SDS) solution with 200 mM boric acid (pH 8.5) over periods lasting from two days to two months, depending on sample size. Solutions were changed bi-weekly. During the final incubation period in an SDS / PBST (0.1% TritonX) 1:1 mixture, the nuclear marker DRAQ5 (1:500) was added (>24 h). After washing twice with PBST (2 d), samples were incubated (>24 h; 37 °C) in refractive index matching solution (RIMS80) containing 80% Nycodenz, 20 mM PBS^−/−^, 0.1% Tween 20, and 0.01% sodium acid. Cleared samples were imaged using a Leica TCS SP8 DLS confocal microscope, equipped with 552 and 633 nm diode lasers at 20× magnification (HC PL APO 20x/0.75 IMM CORR CS2; Leica Microsystems). Rendering and three-dimensional (3D) reconstruction of fluorescence images was performed using Imaris 9 microscopy image analysis software (Bitplane, Zurich, Switzerland).

### Fluorescence Ca^2+^ imaging

#### In situ imaging.

For semi-confocal (pinhole: 5–6 Airy units) live-cell imaging in acute seminiferous tubule slices, tissue sections were either bulk-loaded with Cal-520/AM in the dark (36 µM, **S**_**2**_; 30 min; RT) or, in case of reporter animals expressing GCaMP6f, directly used. After bulk loading, slices were washed (3× ; **S**_**1**_), transferred to a recording chamber, and imaged at either 20× , 25× , or 40× magnification (HC PL APO 20× /0.80 DRY, HCX PL APO CS 20×/0.75 DRY UV, HC PL APO 25×/0.95 W, or HC PL APO 40× /1,30 OIL; Leica Microsystems) with inverted (DMi8, TCS SP5, or TCS SP8 STED; Leica Microsystems) or upright (TCS SP8 MP; Leica Microsystems) microscopes, using built-in confocal scanners (Leica Microsystems) or a multi-beam confocal system (VT-HAWK; VisiTech Int., Sunderland, UK). Cal-520 or GCaMP6f were excited by a 488 nm laser (laser power <20%). Images were acquired at 1–2 Hz using standard PMT or HyD detectors or a cooled EM-CCD camera (Image EM 9100-13, Hamamatsu photonics, Hamamatsu, Japan) controlled by Las X, LAS AF (Leica Microsystems), or VoxCell Scan (VisiTech International) software, respectively.

#### In vivo imaging.

For double-positive adult male offspring of SMMHC-CreER^T2^ × Ai95D mice, we administered tamoxifen (75 mg tamoxifen kg^−1^ body weight) via daily intraperitoneal injections for 5 consecutive days. Animals were closely monitored for any adverse reactions to the treatment. Experiments were performed 2–5 weeks after the first injection*.* For surgery of these (SMMHC-CreER^T2^ × Ai95D) as well as Stra8-Cre × Ai95D and AMH-Cre × Ai95D mice, animals were anesthetized with ketamine–xylazine–buprenorphine (100 mg kg^−1^, 10 mg kg^−1^, 0.05–0.1 mg kg^−1^, respectively; Reckitt Benckiser Healthcare, UK). First, we made an incision next to the *linea alba* in the hypogastric region, followed by a 5 mm incision into the peritoneum. One testis was gently lifted from the abdominal cavity. Its *gubernaculum* was cut and the testis—with the spermatic cord, its blood vessels and *vas deferens* still intact—was transferred to a temperature-controlled imaging chamber filled with extracellular solution (**S**_**1**_; 35 °C), mounted on a custom-designed 3D printed *in vivo* stage [30]. Throughout each experiment, vital signs (heartbeat, blood oxygen level, breathing rhythm) were constantly monitored and recorded (breathing). Moreover, we routinely checked unobstructed blood flow within testicular vessels during experiments. To avoid movement artifacts, the tunica was glued to two holding strings using histoacryl tissue adhesive. After surgery, anesthesia was maintained by constant isoflurane inhalation (1%–1.5% in air). Multiphoton time-lapse intravital imaging was performed using a Leica TCS SP8 MP microscope (Leica Microsystems). Images were acquired at ~2 Hz frame rate using an HCX IRAPO L25×/0.95 W objective (Leica Microsystems) at 930 nm excitation wavelength. After short-pass filtering (680 nm cut-off), light emission was captured by non-descanned (external) hybrid detectors. Bandpass filters isolated GCaMP6f fluorescence (BP 525/50 nm) from autofluorescence (BP 585/40 nm).

Individual cells were identified by their basal GCaMP6f fluorescence as well as by their position and shape as discerned by tissue autofluorescence. Given some ‘leaky’ reporter protein expression in offspring from Stra8-Cre × Ai95D mice (see Discussion), we carefully categorized premeiotic germ cells based on (*i*) their location within the seminiferous epithelium, (*ii*) their epithelial position close to the basal lamina, and (*iii*) their spherical shape.

Experiments investigating endocrine effects on Sertoli cell Ca^2+^ signaling followed a standard experimental protocol: data was acquired from a given field-of-view as 15 min sequences at ~1 h intervals (mean interval 58.4 ± 16.6 min). After control recordings under physiological conditions, we injected either LH or FSH and resumed imaging after 31.4 ± 9.7 min (FSH) or 23.3 ± 9.9 min (LH), respectively.

### Data analysis

All data were obtained from at least three independent experiments performed on at least three days. Individual numbers of cells/tubules/experiments (*n*) are denoted in the respective figures and/or captions as well as in S1 Table. If not stated otherwise, results are presented as means ± SD. Statistical analyses were performed using Mann–Whitney *U* tests, Wilcoxon signed-rank tests, Kruskal–Wallis with *post hoc* Dunn tests and Benjamini–Hochberg-correction, or Fisher´s exact test (as dictated by data distribution and experimental design). Tests and corresponding *p*-values that report statistical significance (≤0.05) are individually specified in captions, further details on statistics are listed in S1 Data. If *p*-values are below 10^−4^, they are reported as *p* < 0.0001 in captions and as exact values in S1 Data. Data were analyzed offline using IGOR Pro 8 & 9 (WaveMetrics, Portland, USA), ImageJ/Fiji (Wayne Rasband, NIH), Excel 2019 (Microsoft, Seattle, WA), and Leica LAS AF & LAS X (Leica Microsystems) software. Additional analysis was performed using custom-written code in MATLAB (The MathWorks, Natick, MA) and Python (Python Software Foundation, Wilmington, USA).

For *post-hoc* categorization of recorded tubules into three groups—i.e., putative early (I‒V), middle (VI‒VIII), and late (IX‒XII) spermatogenic stages [3]—we assessed the absence or presence of late spermatids and, if present, we evaluated their distribution, density, and morphology. With spermiation occurring at stage VIII (Fig 1A), the late stages IX to XII are characterized by absence of late spermatids from the epithelium [48]. Immediately prior to and during spermiation (i.e., in middle stages VI‒VIII), hook-like apical TBCs are formed in preparation of sperm release and late elongated spermatids are densely packed in characteristic adluminal clusters [76]. In the early stages I‒V, elongated spermatids are less condensed and scattered more loosely within the apical seminiferous epithelium [3]. These anatomical characteristics can be inferred from confocal z-stack imaging of epithelial nuclei. We trained a machine learning algorithm (Cellpose-SAM [77]) to identify elongated spermatid nuclei using randomly selected images that were manually segmented (Matlab Image Segmenter toolbox). Next, we ran the algorithm on 30 z-stacks, reconstructed spermatids from the resulting masks and plotted a ‘five-nearest-neighbors’ distribution using Imaris software. The result showed a clear bimodal distribution. For validation, we then categorized z-stacks as either early or middle stage by visual inspection. This labeling matched the algorithmic bimodal distribution with 87% (26 out of 30) accuracy. Yet, to err on the side of caution, we limited stage categorization to middle and late stages. We reason that, given the more diffuse distribution and less strong condensation of spermatid nuclei, putative early stage tubules are harder to identify unequivocally.

For quantitative analysis of time-lapse recordings in tubule slices, images were registered to their respective first image frame at time point, using the Lucas–Kanade algorithm [78] (IAT toolbox for Matlab), resulting in stabilized recordings without movement. Regions of interest (ROIs) were defined manually at and superimposed onto all subsequent images of the stabilized recording. For each ROI at each time point, the mean fluorescence signal was computed and normalized with respect to a baseline before signal onset, computing the intensity change for the *i*th time point as $\frac{F_{t}-F_{baseline}}{F_{t}}$. For clarity, linear baseline shifts were corrected if necessary.

For slice experiments investigating potential effects of ORAI inhibition or FSH exposure, images were registered to a sequence-specific reference frame (fr_50_), acquired when 50% of recording time had elapsed. Registration was implemented in Python using the SimpleITK package with SimpleElastix extension [79,80]. Based on the B-Spline method, we selected a deformable registration approach, using the adaptive stochastic gradient descent optimizer and the advanced Mattes mutual information similarity metric. We employed 1,000 iterations with 2048 randomized spatial samples and a multi-resolution approach with six grid spacing scheduler levels {64, 32, 16, 8, 2, 1}. fr_50_ served as the fixed image; all other frames were used as moving images and individually registered to this reference. Registration was parallelized using the parfor package and implemented for batch processing of a list of videos.

For analysis of two-photon *in vivo* data acquired from double-positive offspring of Stra8-Cre × Ai95D mice, we used the MATLAB implementation of DeepInterpolation [81], a machine learning algorithm to remove random shot noise, for image restoration. The network was trained on noisy raw data for each video. All quantitative analyses used raw (non-denoised) intensity data. For analysis of all *in vivo* data, we employed custom sets of ImageJ macros utilizing built-in Fiji-ImageJ functions [82,83]. To identify and isolate seminiferous tubule areas and periods characterized by spontaneous contractions and other substantial movements, we applied *optical flow* analysis to the background/autofluorescence channel (BP 585/40 nm). The resulting additional ‘movement channel’ allowed us to cut/ignore affected regions and frames and, thus, avoid movement artifacts in the analysis. ROIs encompassing spermatogonia and TPCs were defined manually. Sertoli cell ROIs were automatically segmented using the *transient signal enhancer* script. False segmentations were removed upon subsequent visual inspection. For each ROI and frame, fluorescence intensity was integrated from raw data. To label hotspots we created time-lapse maximum projections and defined hotspots as clusters of activity that meet the following criteria: (*i*) areas of high activity span at least 20,000 µm^2^ for adult, or 18,000 µm^2^ for juvenile tubules (i.e., >5.8%/>5.2% of the field-of-view), (*ii*) hotspots include at least five active ROIs, and (*iii*) active ROIs comprise at least 3% of each hotspot (which always includes the entire visible tissue in either a real or ‘virtual’ tubular cross-section).

Analysis of original intensity *versus* time data traces was performed in Python using custom code. Traces were baseline-corrected using the *noise_median* function (Pybaselines package) with default parameters. Frames/regions contaminated by contractions or other movement artifacts were removed, including the preceding and subsequent 40 frames. Next, time series data was z-normalized. We employed the *find_peaks* function (SciPy [84]; prominence = 0.27/threshold = 0.08 for TPCs, prominence = 4 for Sertoli cells) for peak detection. These empirically determined parameters were evaluated based on sensitivity and positive predictive value. Traces were either analyzed over the entire recording length (no interruptions by contractions/movements) or within segments flanked by such movements. Active periods were measured from first to last peak, and event frequency was calculated within such periods. Ca^2+^ elevations that lasted for more than 3 min were categorized as persistent signals. In slice experiments investigating potential effects of ORAI inhibition or FSH exposure, Ca^2+^ signal kinetics were analyzed using custom Python code. After baseline correction (Pybaselines) we calculated baseline mean and SD values. Events were defined based on a mean + 3 × SD threshold and results were manually curated.

For signal correlation analysis, each ROI was assigned to its tubule of origin. ROI pairs assigned to different tubules were classified as *inter*-tubular pairs, while pairs from the same tubule were classified as *intra*-tubular pairs. All traces were corrected using a median-noise filter. To align trace pairs with congruent temporal structures, we removed single movement periods from both corresponding traces.

All custom code (Python, MATLAB, Fiji scripts) is available in a zenodo repository (https://doi.org/10.5281/zenodo.20938794). All parameters and preprocessing steps were chosen to optimize signal quality as well as reproducibility, and are detailed in the code provided.

## Supporting information

S1 Table Statistical transparency table. Summary of sampling, measurement, and analysis parameters.
(PDF)

S1 Fig TPCs and Sertoli cells are affected by extracellular Ca^2+^ removal on different time scales. (**A**) Semi-confocal microscopy of GCaMP6f fluorescence in isolated tubules from SMMHC-Cre × Ai95D (top) and AMH-Cre × Ai95D (bottom) mice. (**B**) Original recordings from two representative cells show changes in Ca^2+^ concentration (Δ*F*/*F*_0_) over time. Traces depict signals prior to and after treatment with reduced extracellular Ca^2+^. (**C**) Quantification of residual cellular activity in TPCs *versus* Sertoli cells during treatment, derived from recordings as shown in (B). Asterisk indicates statistical signiﬁcance (*p*^1^ < 0.0001; Fisher’s exact test). The underlying numerical data for this figure is detailed in S1 Data.
(TIF)

S2 Fig In juvenile testes, transient Sertoli cell activity is spatially confined and occurs in dynamic hotspots. (**A**) Representative multiphoton *in vivo* images from juvenile AMH-Cre × Ai95D mice depicting either a single-frame (**A**_***I***_) or the corresponding time-lapse maximum projection (**A**_***II***_) that identifies hotspots of Sertoli cell activity (white asterisk). Individual tubules outlined by dotted white lines. Pseudocolors (inferno color map) indicate relative Ca^2+^ concentration. Note that tubules are smaller than in adults. (**B**) Paired dot plot comparing activity in adluminal *versus* basal areas. Asterisk indicates statistical signiﬁcance (*p* = 5.9 × 10^−5^; two-tailed Wilcoxon signed-rank test). (**C**) Wheel chart quantifying *in vivo* observations of hotspot activity in juvenile mice. (**D**) Dot and violin plot depicting absolute hotspot size. (**E**) Long-term recurrent recordings from the same fields-of-view reveal dynamic switches between hotspot activity and quiescent states. Line colors indicate sustained (orange), gain (yellow), multiple switches (red), or loss (purple) of hotspot activity. The underlying numerical data for this figure is detailed in S1 Data.
(TIF)

S3 Fig LH affects adult Sertoli cell signaling *in vivo.* (**A**) Schematic illustrating the experimental strategy. (**B**) Paired dot plots comparing transient activity in adluminal *versus* basal Sertoli cell areas under control conditions (gray) as well as after systemic injection of LH (blue). (**C**&**D**) Dot and violin plots depicting absolute (**C**) and relative (**D**) hotspot size. Color code as in (B). Black dots in (D) represent means, diamonds display median values (mean ± SD = 0.54 ± 0.2 (control), 0.58 ± 0.16 (LH); median = 0.54 (control), 0.61 (LH)). Absolute values (C) are compared before versus after treatment (lines label paired regions). (**E**) Long-term recurrent recordings from the same fields-of-view under control conditions ((**E_*I*_**); see also Fig 5D) as well as before *versus* after LH (**E**_***II***_) treatment. Line colors indicate sustained (orange), gain (yellow), multiple switches (red), or loss (purple) of hotspot activity. Note that data is only included if a tubule displayed hotspot activity at least once and if at least one pre- and one post-treatment period had been measured per field-of-view. (**F**) Percentage of hotspot occurrence during pre- *versus* post-treatment periods. Note that all tubules are included, i.e., independent of the inclusion criteria described in (E). (**G**) Wheel charts quantifying the occurrence of persistent Ca^2+^ elevations under control conditions and after LH treatment, respectively. The underlying numerical data for this figure is detailed in S1 Data.
(TIF)

S4 Fig FSH effects on Sertoli cell *in vivo* signaling in juvenile mice. (**A**) Schematic illustrating the experimental strategy. (**B**) Paired dot plots comparing transient activity in adluminal *versus* basal Sertoli cell areas under control conditions (gray) as well as after systemic injection of FSH (red). (**C**) Dot and violin plots depicting hotspot size. Color code as in (B). Values are compared before *versus* after treatment (lines label paired regions). (**D**) Long-term recurrent recordings from the same fields-of-view under control conditions (**D**_***I***_) as well as before *versus* after FSH (**D**_***II***_) treatment. Line colors indicate sustained (orange), gain (yellow), multiple switches (red), or loss (purple) of hotspot activity. Note that data is only included if a tubule displayed hotspot activity at least once and if at least one pre- and one post-treatment period had been measured per field-of-view. (**E**) Percentage of hotspot occurrence during pre- *versus* post-treatment periods. Note that all tubules are included, i.e., independent of the inclusion criteria described in (D). Asterisk indicates statistical signiﬁcance (*p*^1^ = 0.00002; Fisher’s Exact test). (**F**) Wheel charts quantifying the *in vivo* occurrence of persistent Ca^2+^ elevations under control conditions *versus* FSH treatment, respectively. The underlying numerical data for this figure is detailed in S1 Data.
(TIF)

S1 Movie Spontaneous Ca^2+^ signals in a wild-type mouse seminiferous tubule. Semi-confocal imaging of cellular *in vitro* activity in an isolated seminiferous tubule from an adult male C57BL/6 mouse bulk-loaded with Cal-520/AM. Pseudocolors (inferno color map) indicate relative Ca^2+^ concentration.
(MP4)

S2 Movie SMMHC-CreER^T2^ mice allow inducible expression of genetically encoded fluorescent reporter proteins in TPCs. After tamoxifen injection, SMMHC-CreER^T2^ × Ai14D male offspring express tdTomato (magenta) in both TPCs and vascular smooth muscle cells. Video shows the 3D reconstruction of a cleared [85] testis sample with nuclei labeled by DRAQ5 (cyan). * = small blood vessels.
(MP4)

S3 Movie AMH-Cre mice allow Sertoli cell-specific expression of genetically encoded fluorescent reporter proteins. AMH-Cre × Ai14D male offspring express tdTomato (magenta) in Sertoli cells. Video shows the 3D reconstruction of a cleared [85] testis sample with nuclei labeled by DRAQ5 (cyan).
(MP4)

S4 Movie Stra8-Cre mice allow expression of genetically encoded fluorescent reporter proteins in spermatogonia. Stra8-Cre × Ai14D male offspring express tdTomato (magenta) in germ cells, mainly spermatogonia. Video shows the 3D reconstruction of a cleared [85] testis sample from juvenile mouse (P21) with nuclei labeled by DRAQ5 (cyan).
(MP4)

S5 Movie Transient Ca^2+^ signals in adluminal Sertoli cell compartments. Multiphoton *in vivo* imaging of Sertoli cell activity in adult male AMH-Cre × GCaMP6f mice. Pseudocolors (inferno color map) indicate relative Ca^2+^ concentration.
(MP4)

S6 Movie Persistent Ca^2+^ signals in Sertoli cells. Multiphoton *in vivo* imaging of Sertoli cell activity in adult male AMH-Cre × GCaMP6f mice. Pseudocolors (inferno color map) indicate relative Ca^2+^ concentration.
(MP4)

S7 Movie Intracellular Ca^2+^ waves in Sertoli cells. Semi-confocal imaging of Sertoli cell *in vitro* activity in an isolated seminiferous tubule section from an adult male AMH-Cre × GCaMP6f mouse. Pseudocolors (inferno color map) indicate relative Ca^2+^ concentration.
(MP4)

S1 Data Excel file containing the numerical data underlying all figures in the main manuscript and supporting information. Each sheet corresponds to a specific figure.
(XLSX)

## Abbreviations

BTB: blood-testis barrier
CPA: cyclopiazonic acid
CV: coefficient of variation
ER: endoplasmic reticulum
FELASA: Federation of European Laboratory Animal Science Associations
FSH: follicle-stimulating hormone
IP_3_: inositol 1,4,5-trisphosphate
IP3R1: IP_3_ receptor type 1
LH: luteinizing hormone
PFA: paraformaldehyde
ROI: region of interest
RT: room temperature
SDS: sodium dodecyl sulfate
SERCA: sarco/endoplasmic reticulum Ca^2+^-ATPase
SOCE: store-operated Ca^2+^ entry
STIM1: stromal interaction molecule 1
TBC: tubulobulbar complex
TPC: testicular peritubular cell
5ppase: 5-phosphatase
