# Supplementary material for: Cell type-specific Ca2+ signals govern mouse seminiferous tubule physiology
Source: PLoS Biol. 2026 Jul 24;24(7):e3003910. doi: 10.1371/journal.pbio.3003910 (PMC13399359; doi:10.1371/journal.pbio.3003910)
Supplement: S1 Table — Summary of sampling, measurement, and analysis parameters. (PDF) [file pbio.3003910.s001.pdf]

Supplementary Table 1: Statistics and Analysis Parameters

Figure	Nₒ animals	Nₒ tubules	Nₒ cells	Nₒ tubules/ animal (mean ± SD)	Nₒ cells/ animal (mean ± SD)	Length of imaging sequence	Nₒ imaging sequences/ tubule (in vitro) or area (in vivo)
1 d,e,f,h,l,j	25	143	1786*	5.1 ± 2.0 (range 1 - 10)	71.3 ± 28.7 (range 3 – 164)	600 s	1
2 d low Ca^{2+}, 3 & 5 min preinc.	4	8	32	2 ± 0.7 (range 1 – 3)	8 ± 2.2 (range 5 – 11)	250 s	3
2 d low Ca^{2+}, 13 min preinc	3	6	27	2 ± 1.4 (range 1 – 4)	9 ± 8.5 (range 2 – 21)	250 s	3
2 d thapsigar gin	4	8	42	2.0 ± 0.7 (range 1 – 3)	10.5 ± 2.7 (range 6 – 13)	250 s	2
2 d CPA	4	10	41	2.5 ± 1.1 (range 1 – 4)	10.25 ± 6.1 (range 4 – 19)	250 s	3
2 f,g GSK	6	42	807*	7 ± 2.58 (range 4-11)	134.5 ± 60.1 (range 43-234)	600 s	2
2 k,l ctrl 1	10	50	319	5 ± 2.6 (range 1 – 10)	31.9 ± 21 (range 4 – 78)	500 s	1
2 k,l ctrl 2	3	11	26	3.7 ± 1.9 (range 1 – 5)	8.7 ± 2.4 (range 7 – 11)	500 s	1
2 j,k,l 5ppase	3	17	34	5.7 ± 1.2 (range 4 – 7)	11.3 ± 2.1 (range 9 – 14)	500 s	1
2 j,k,l IP3RKD	4	18	46	4.5 ± 2.8 (range 2 – 9)	11.5 ± 6.5 (range 6 – 21)	500 s	1
3 d pmGC	9	24	10*	2.4 ± 1.7 (range 1 – 6)	2 ± 1.5* (range 0 – 5)	600 s	1
3 d – j TPC	6	86	113*	6.5 ± 2.3 (range 3 – 9)	18.8 ± 8.0 (7 – 25)	600 s	1
3 d – j SC	6	42	173*	5.2 ± 2.6 (range 1 – 8)	29.2 ± 14.7 (range 6 – 44)	600 s	1
4 a,d,f,g pmGC	5	76	20*	4.4 ± 0.8 (range 3 – 5)	2.1 ± 1.0 (range 1 – 4)	900 s	1.5 ± 0.5 (range 1 – 2)
4 b,e, TPC	15	116	183*	4.6 ± 1.3 (range 2 – 6)	7.8 ± 3.0 (range 3 – 15)	900 s	1.3 ± 0.5 (range 1 – 2)
4, c,e,f,g,h SC	5	340	1682*	5.0 ± 2.5 (range 1 – 10)	101.7 ± 70.1 (range 2 – 307)	900 s	1
4 i SC	8	90	N/A	11.3 ± 4.4 (range 7 – 20)	N/A	900 s	4.5 ± 0.9 (range 1 – 5)
5 b,c,d	5	42	N/A	8.4 ± 0.9 (range 7 – 9)	N/A	900 s	3.5 ± 0.5 (range 1 – 4)
5 f, h	15	96	1168*	4.95±1.96 (range 2-10)	58.15±32.96 (19-165)	600s	1
5 i,j,k	11	29	381*	1.93±0,85 (range 1-4)	25.4±17.96 (range 4-70)	600s	1
6 b	13	26	761*	2.6 ± 0.9 (range 1 – 3)	58.5 ± 68.9 (range 5 – 246)	900 s	1.3 ± 0.6 (range 1 – 3)
6 f	6	6	25	1 ± 0 no range	4.2 ± 1.3 (range 3 – 6)	600 s	1
7 c	15	116	183*	4.6 ± 1.3 (range 2 – 6)	7.8 ± 3.0 (range 3 – 15)	900 s	1.3 ± 0.5 (range 1 – 2)
7 d	5	340	1682*	5.0 ± 2.5 (range 1 – 10)	101.7 ± 70.1 (range 2 – 307)	900 s	1
7 e	8	90	169*	11.3 ± 4.4 (range 7 – 20)	21.1 ± 24 (0 – 59)	900 s	4.5 ± 0.9 (range 1 – 5)

Figure	N_o animals	N_o tubules	N_o cells	N_o tubules/ animal (mean ± SD)	N_o cells/ animal (mean ± SD)	Length of imaging sequence	N_o imaging sequences/ tubule (in vitro) or area (in vivo)
8 b ctr	13	24	482*	2.6 ± 1.3 (range 1 – 6)	58.5 ± 68.9 (range 5 – 246)	900 s	1
8 b FSH	3	6	190*	1.7 ± 1.2 (range 1 – 3)	64.3 ± 73.4 (range 11 – 148)	900s	1
8 d,e,f$_{I}$,g ctr	5	42	N/A	8.4 ± 0.9 (range 7 – 9)	N/A	900 s	3.5 ± 0.5 (range 1 – 4)
8 d,e,f$_{III}$,g FSH	6	47	N/A	7.2 ± 1.8 (range 4 – 9)	N/A	900 s	2.7 ± 0.7 (range 2 – 4)
8 i,j,k ctr	5	23	185*	4.6 ± 1.9 (range 2 – 9)	37.0 ± 3.6 (range 31 – 41)	600s	1
8 i,j,k FSH	8	42	466*	4.7 ± 2 (range 2 – 9)	51.8 ± 44.7 (range 12 – 160)	600s	1
8 l in vivo ctr	8	90	N/A	11.3 ± 4.4 (range 7 – 20)	N/A	900 s	4.5 ± 0.9 (range 1 – 5)
8 l in vivo FSH	7	54	N/A	7.7 ± 0.95 (range 6 – 9)	N/A	900 s	2.6 ± 1. 2 (range 1 – 5)
8 l in vitro ctr	15	99	N/A	5.0 ±2.0 (range 2 – 10)	N/A	600s	1
8 l in vitro FSH	8	43	N/A	4.8 ± 1.9 (range 2 – 9)	N/A	600s	1
S1 c	5	10	28	2.0 ± 0.7 (range 1 – 3)	5.6 ± 2.1 (range 4 – 9)	120 s	1
S1 c	5	15	78	2.1 ± 0.9 (range 1 – 3)	11.1 ± 7.5 (range 2 – 21)	120 s	1
S2 b	11	45	645*	4.1 ± 2.4 (range 1 – 8)	58 ± 55 (range 155 – 6)	900 s	1
S2 c,d,e	4	44	N/A	11.0 ± 2.9 (range 8 – 15)	N/A	900 s	3.2 ± 0.9 (range 2 – 4)
S3 b ctr	13	24	482*	2.6 ± 1.3 (range 1 – 6)	58.5 ± 68.9 (range 5 – 246)	900 s	1
S3 b LH	3	4	77*	1.3 ± 0.6 (range 1 – 2)	26.3 ± 24.4 (range 5 – 53)	900 s	1
S3 c,d,e,f ctr	5	42	N/A	8.4 ± 0.9 (range 7 – 9)	N/A	900 s	3.5 ± 0.5 (range 1 – 4)
S3 c,d,e,f LH	7	51	N/A	7.3 ± 2.3 (range 7 – 9)	N/A	900 s	2.7 ± 0.5 (range 2 – 4)
S3 g ctr	8	90	N/A	11.3 ± 4.4 (range 7 – 20)	N/A	900 s	4.5 ± 0.9 (range 1 – 5)
S3 g LH	8	61	N/A	7.6 ± 2.1 (range 4 – 10)	N/A	900 s	2.8 ± 1. 4 (range 1 – 6)
S4 b ctr	11	45	N/A	4.1 ± 2.4 (range 1 – 8)	N/A	900 s	1
S4 c,d,e ctr	4	44	N/A	11.0 ± 2.9 (range 8 – 15)	N/A	900 s	3.2 ± 0.9 (range 2 – 4)
S4 c,d,e FSH	6	68	N/A	11.3 ± 3.5 (range 8 – 17)	N/A	900 s	2.6 ± 0.6 (range 2 – 3)
S4 f ctr	6	99	N/A	16.5 ± 7.1 (range 11 – 29)	N/A	900 s	3.75 ± 2 (range 1 – 9)
S4 f FSH	6	69	N/A	12.66 ± 3.5 (range 9 – 17)	N/A	900 s	4.24 ± 1.4 (range 2 – 6)

N/A not applicable

* only active cells

Fig. 3 pmGCs: most experiments without any active cells (18/24), no analysis with ROIs in silent tubules
Fig. 4 pmGCs: most ROIs without activity, all measured ROIs per animal: 27.8 ± 9.8 (range 7 – 42)
