# Supplementary figures and images for: Cell type-specific Ca2+ signals govern mouse seminiferous tubule physiology
Source: PLoS Biol. 2026 Jul 24;24(7):e3003910. doi: 10.1371/journal.pbio.3003910 (PMC13399359; doi:10.1371/journal.pbio.3003910)

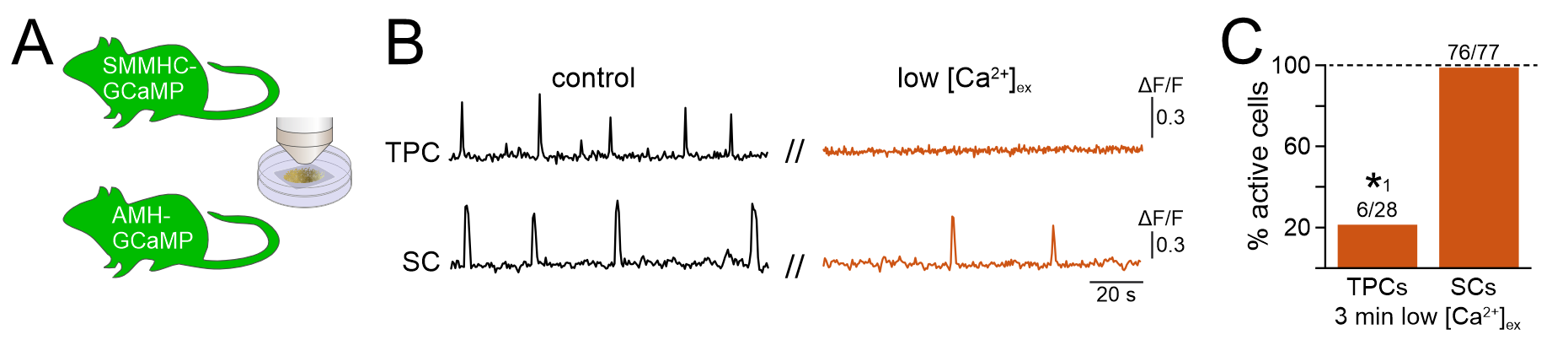

Supplement: S1 Fig — (A) Semi-confocal microscopy of GCaMP6f fluorescence in isolated tubules from SMMHC-Cre × Ai95D (top) and AMH-Cre × Ai95D (bottom) mice. (B) Original recordings from two representative cells show changes in Ca2+ concentration (ΔF/F0) over time. Traces depict signals prior to and after treatment with reduced extracellular Ca2+. (C) Quantification of residual cellular activity in TPCs versus Sertoli cells during treatment, derived from recordings as shown in (B). Asterisk indicates statistical signiﬁcance (p1 < 0.0001; Fisher’s exact test). The underlying numerical data for this figure is detailed in S1 Data. (TIF) [file pbio.3003910.s002.tif]

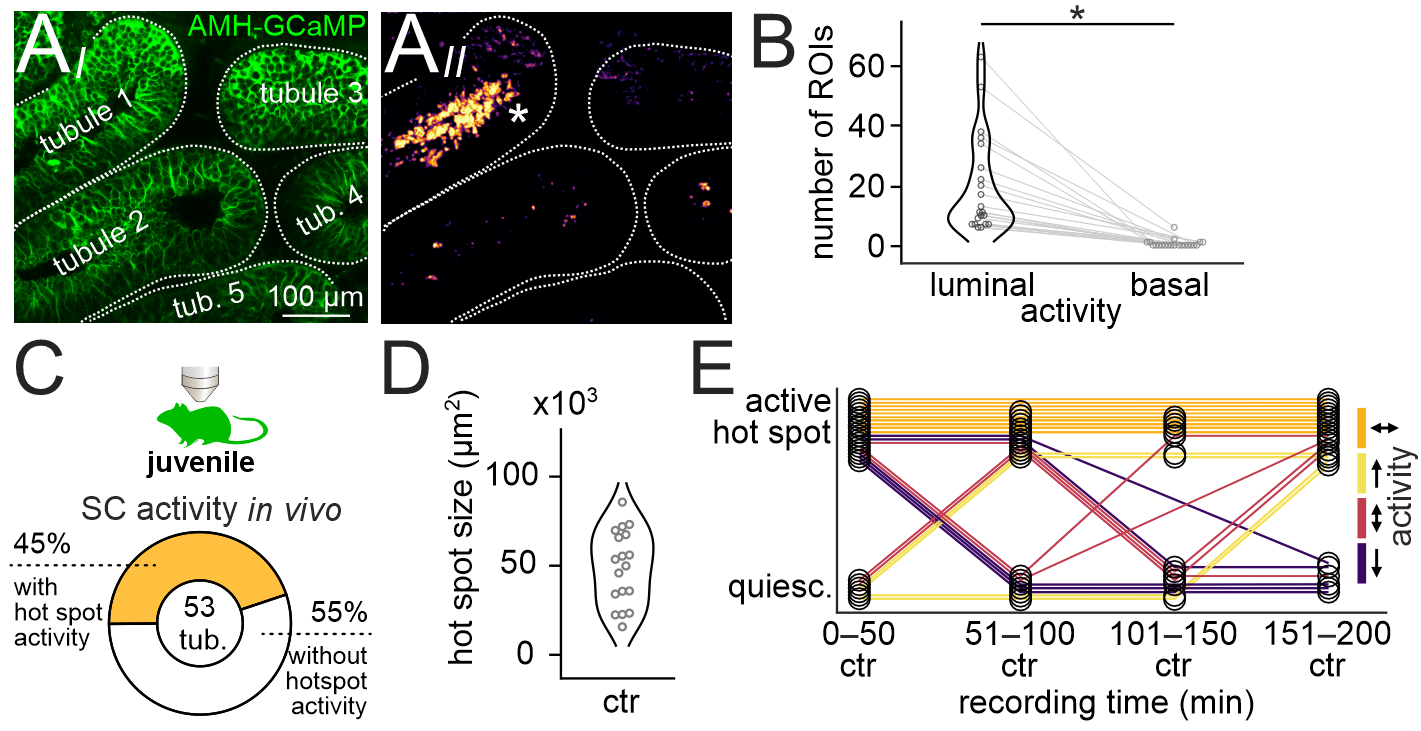

Supplement: S2 Fig — (A) Representative multiphoton in vivo images from juvenile AMH-Cre × Ai95D mice depicting either a single-frame (AI) or the corresponding time-lapse maximum projection (AII) that identifies hotspots of Sertoli cell activity (white asterisk). Individual tubules outlined by dotted white lines. Pseudocolors (inferno color map) indicate relative Ca2+ concentration. Note that tubules are smaller than in adults. (B) Paired dot plot comparing activity in adluminal versus basal areas. Asterisk indicates statistical signiﬁcance (p = 5.9 × 10^−5^; two-tailed Wilcoxon signed-rank test). (C) Wheel chart quantifying in vivo observations of hotspot activity in juvenile mice. (D) Dot and violin plot depicting absolute hotspot size. (E) Long-term recurrent recordings from the same fields-of-view reveal dynamic switches between hotspot activity and quiescent states. Line colors indicate sustained (orange), gain (yellow), multiple switches (red), or loss (purple) of hotspot activity. The underlying numerical data for this figure is detailed in S1 Data. (TIF) [file pbio.3003910.s003.tif]

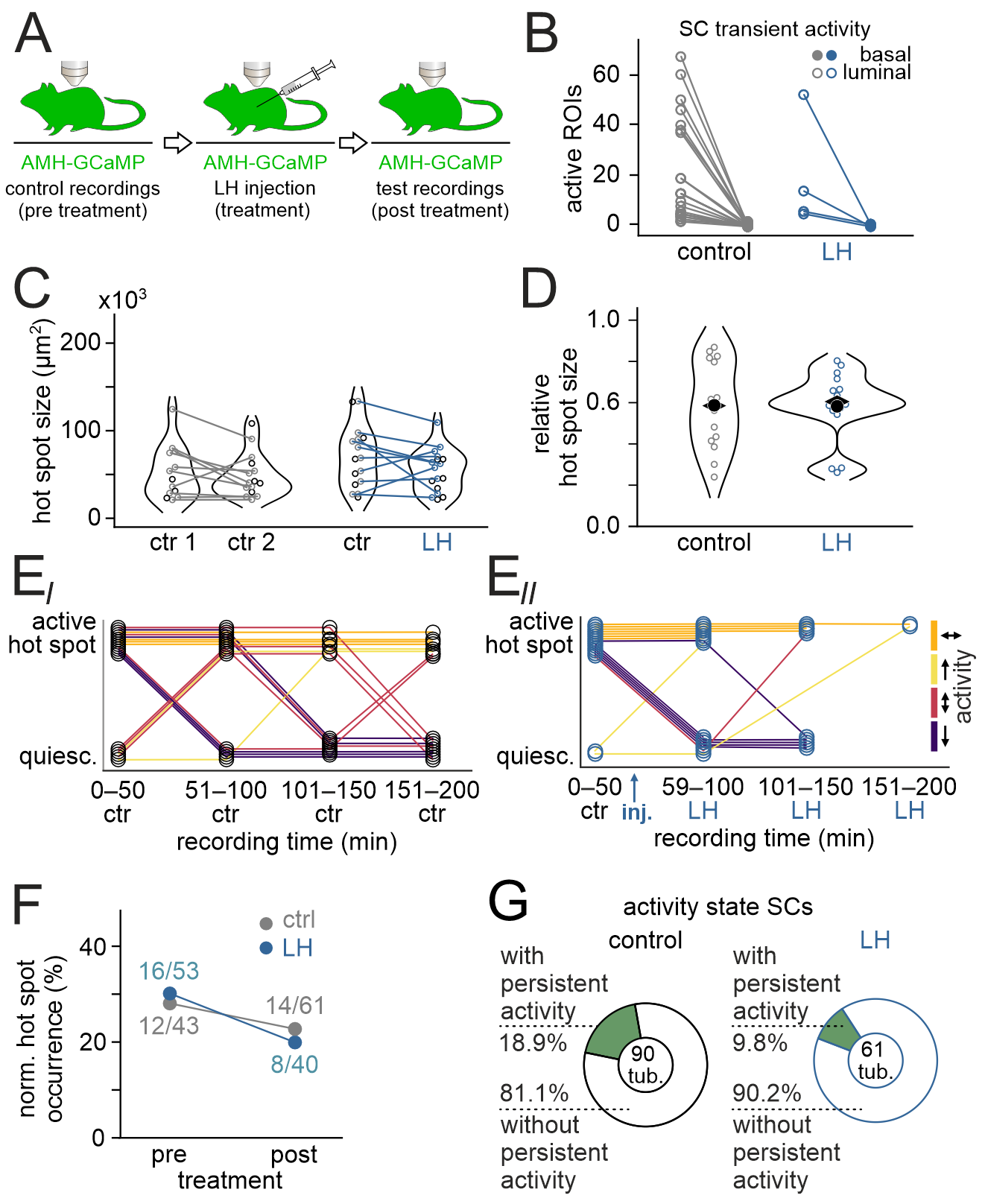

Supplement: S3 Fig — (A) Schematic illustrating the experimental strategy. (B) Paired dot plots comparing transient activity in adluminal versus basal Sertoli cell areas under control conditions (gray) as well as after systemic injection of LH (blue). (C&D) Dot and violin plots depicting absolute (C) and relative (D) hotspot size. Color code as in (B). Black dots in (D) represent means, diamonds display median values (mean ± SD = 0.54 ± 0.2 (control), 0.58 ± 0.16 (LH); median = 0.54 (control), 0.61 (LH)). Absolute values (C) are compared before versus after treatment (lines label paired regions). (E) Long-term recurrent recordings from the same fields-of-view under control conditions ((EI); see also Fig 5D) as well as before versus after LH (EII) treatment. Line colors indicate sustained (orange), gain (yellow), multiple switches (red), or loss (purple) of hotspot activity. Note that data is only included if a tubule displayed hotspot activity at least once and if at least one pre- and one post-treatment period had been measured per field-of-view. (F) Percentage of hotspot occurrence during pre- versus post-treatment periods. Note that all tubules are included, i.e., independent of the inclusion criteria described in (E). (G) Wheel charts quantifying the occurrence of persistent Ca2+ elevations under control conditions and after LH treatment, respectively. The underlying numerical data for this figure is detailed in S1 Data. (TIF) [file pbio.3003910.s004.tif]

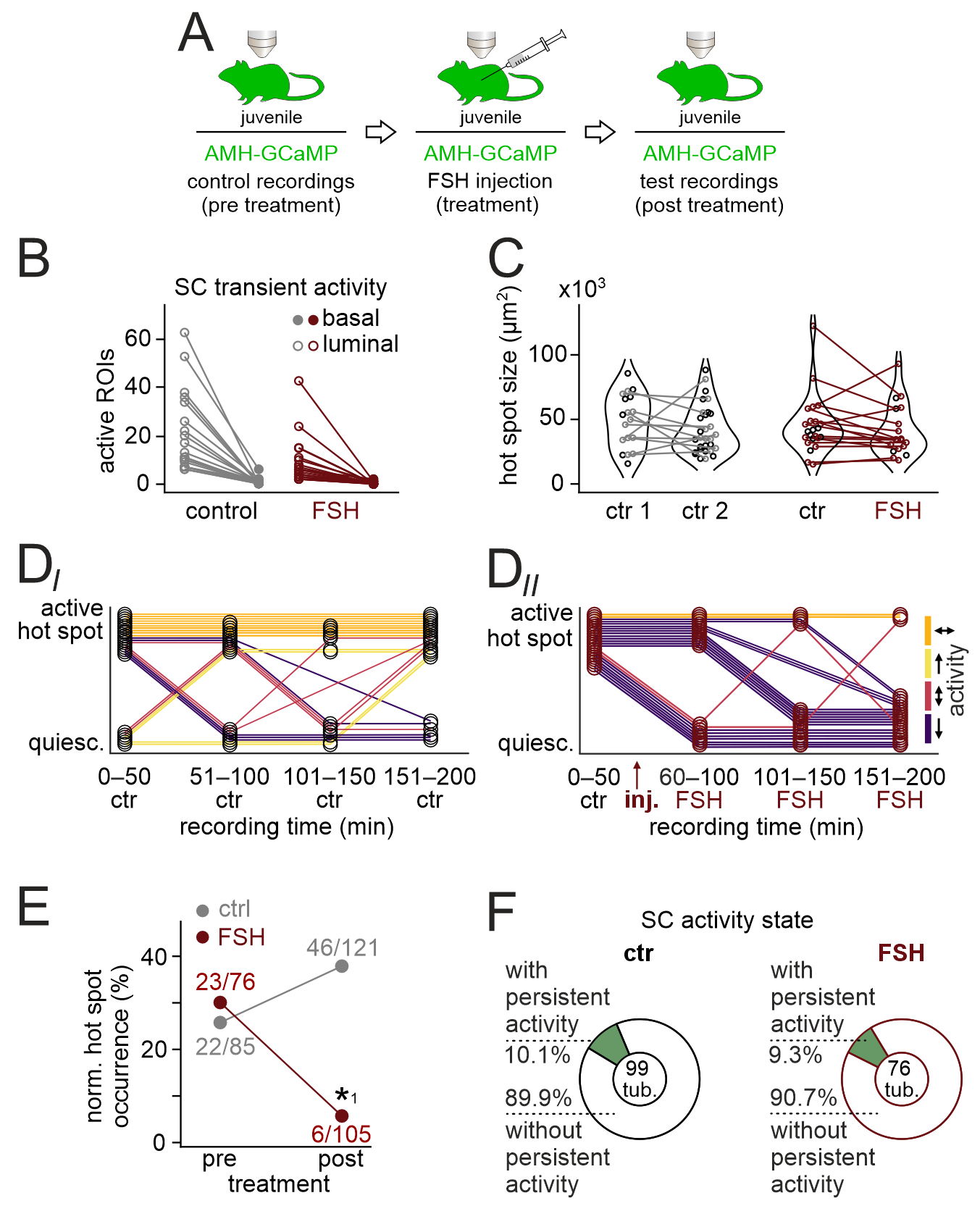

Supplement: S4 Fig — (A) Schematic illustrating the experimental strategy. (B) Paired dot plots comparing transient activity in adluminal versus basal Sertoli cell areas under control conditions (gray) as well as after systemic injection of FSH (red). (C) Dot and violin plots depicting hotspot size. Color code as in (B). Values are compared before versus after treatment (lines label paired regions). (D) Long-term recurrent recordings from the same fields-of-view under control conditions (DI) as well as before versus after FSH (DII) treatment. Line colors indicate sustained (orange), gain (yellow), multiple switches (red), or loss (purple) of hotspot activity. Note that data is only included if a tubule displayed hotspot activity at least once and if at least one pre- and one post-treatment period had been measured per field-of-view. (E) Percentage of hotspot occurrence during pre- versus post-treatment periods. Note that all tubules are included, i.e., independent of the inclusion criteria described in (D). Asterisk indicates statistical signiﬁcance (p1 = 0.00002; Fisher’s Exact test). (F) Wheel charts quantifying the in vivo occurrence of persistent Ca2+ elevations under control conditions versus FSH treatment, respectively. The underlying numerical data for this figure is detailed in S1 Data. (TIF) [file pbio.3003910.s005.tif]
