# Supplementary material for: Cell type-specific Ca2+ signals govern mouse seminiferous tubule physiology
Source: PLoS Biol. 2026 Jul 24;24(7):e3003910. doi: 10.1371/journal.pbio.3003910 (PMC13399359; doi:10.1371/journal.pbio.3003910)
Supplement: Attachment — Submitted filename: Response to Reviewers.pdf [file pbio.3003910.s016.pdf]

Reply to the Referees' Comments

We thank the Expert Referees for critically reading the manuscript and for their highly instructive and thoughtful comments, which helped us create an improved manuscript. Based on the comments of both reviewers as well as the Academic Editor, we have extensively revised our paper, adding new experimental data and analysis (now shown in several new figures and additional panels, i.e. ***new / revised Figs. 1D***&***F***, ***2C***–***G***, ***2J***&***L***, ***4E***&***H***, ***5E***–***K***, ***8H***–***L***, ***S1***, ***S2***, and ***S4***, as well as new supporting ***Table 1*** and new supporting ***Movie M1***).

To address the Experts' comments, we now submit a revised manuscript that extends our findings, as described below in a point-by-point reply (marked in dark blue font) to the reviews (in *italics*).

Comments to referee 1:

Reviewer 1 attests that we "*present an ambitious and technically advanced study examining Ca^{2+} signaling across the three main cellular components of the mouse seminiferous tubule (Sertoli cells, peritubular myoid cells, and premeiotic germ cells) using both acute tubule slices and intravital multiphoton imaging. The manuscript heavily depends on Credriver specificity to assert cell-type-specific Ca^{2+}.*"

This reviewer emphasized that our "*objective is to define cell-type-specific Ca^{2+} "fingerprints" and to suggest how these signatures influence aspects of seminiferous tubule physiology, including endocrine responsiveness, addressing a genuine gap in reproductive physiology: how Ca^{2+} signals are organized within the intact seminiferous epithelium and how these signals differ among supporting cell types' responses. Cytosolic Ca^{2+} levels were measured in three cell types of the mouse seminiferous tubule to better understand the physiological mechanisms regulating mammalian spermatogenesis, which still largely remains a biological black box. Both in vitro and in vivo studies show clear, cell-specific signal patterns that (i) have distinct spatiotemporal signatures, (ii) depend on IP3-mediated Ca^{2+} release from endoplasmic stores, and (iii) are influenced by gonadotropins.*"

The expert continues by stating that "*while the imaging technique is technically proficient and some observations are truly intriguing, the manuscript frequently relies more on interpretation than direct evidence. Many key claims are undermined by methodological constraints, insufficient validation of cell identities, and overambitious claims about what the data can legitimately support. Currently, the manuscript appears to be shaped by the capabilities of the imaging system rather than by considerations of biological importance, causality, or physiological relevance.*" In addition to these general remarks, reviewer 1 outlines several specific points that we need to address.

We thank the reviewer, acknowledge their arguments and, accordingly, we have addressed all these remarks and "*major comments*" in the revised manuscript as outlined below:

1) "*Statistical transparency: For a study that relies heavily on event frequency, duration, and oscillatory behavior, clear information about sampling is crucial. Several analyses would benefit from explicitly reporting: the number of tubules versus the number of cells per animal, variability between animals, the number of signals recorded across how many imaging*

intervals, and the criteria for defining "frequent" versus "sporadic" events. A brief supplemental table could address most of these points."

We thank reviewer 1 for raising this important point and for their insightful suggestion. Accordingly, in a new supporting table (Table 1), we now report (*i*) the exact numbers of cells, tubules, and animals used for sampling, (*ii*) the means, standard deviations, and ranges for cells and tubules per animal, (*iii*) the duration of imaging sequences for each experimental paradigm, (*iv*) the numbers of imaging sequences per tubule (Figs. 1-3) and/or field-of-view (Figs. 4-8), and (*v*) the means, standard deviations, and ranges for number of signals per imaging sequence. We now refer to Table 1 throughout the manuscript wherever relevant (pp. 4 (l. 7), 5 (l. 11), 8 (l. 1), 8 (l. 23), 10 (l. 4), 11 (l. 16), 12 (l. 9), and 13 (l. 5)).

We apologize for not being clear on our definition of *"frequent" versus "sporadic"* events. This categorization is determined by the number of events recorded. When plotting the event number per time period for all cells recorded *in vitro* under control conditions (new Fig. 1D) we note a multimodal distribution. Multipeak fitting identifies the first population as cells exhibiting ≤ 5 Ca^{2+} signals within 10 min. We categorize this group of cells as sporadically active, whereas those displaying ≥ 6 Ca^{2+} transients are labeled as frequently active. In the original manuscript, we also showed individual peak distance distributions (former Fig. 1f). We now replaced this result by plotting a measure of signal regularity. For all cells, we plotted their inter-event intervals' coefficient of variation (CV). In the revised manuscript, we now show this CV distribution as new Fig. 1F. This violin plot depicts a broad, though apparently normal distribution of individual CVs. The lack of distinct populations and the relatively high mean value suggest that rather few cells, if any at all, display tightly orchestrated oscillatory signals of high periodicity. This result is relevant since the originally depicted individual peak distance distributions (based on recordings from 62 cells) indicated "*some degree of periodicity*" among more active cells. Now, having analyzed an additional 1,724 cells and using a more meaningful measure (i.e., inter-event interval CV), we revised that statement accordingly.

We added corresponding information to the *Results* text, which now reads (p. 4; ll. 5–11): "*Prolonged (10 min) semi-confocal fluorescence imaging revealed essentially two types of spontaneous cellular activity (Fig 1C; Movie M1). We categorize these patterns (Fig 1D&E; Table 1) according to event number as (i) frequent activity (32% of cells; ≥ 6 events / 10 min), or (ii) sporadic and seemingly random activity (sporadic; 68% of cells; ≤ 5 events / 10 min). Analysis of inter-event interval coefficients of variation (CV; Fig 1F) reveals a broad, apparently normal distribution of individual CVs. The lack of a distinct cell population with low CV values suggests that rather few cells, if any, display highly periodic oscillatory signals*."

Note that, compared to our original manuscript, the numbers / percentages of "frequent" *versus* "sporadic" cells changed. The reason is that we now included data from all new slice experiments performed under control conditions (see our comments to referee 2 and the Academic Editor below). While this considerably improved data robustness, our general findings and interpretations remain the same.

2) *"Cellular Identification and Driver Line Specificity: The manuscript heavily depends on Cre-driver specificity to assert cell-type-specific Ca^{2+} responses. Although AMH-Cre and SMMHC-CreERT2 are generally reliable, the authors acknowledge leakage issues with Stra8-Cre mice. This point is significant and warrants more emphasis. When studying a very sparse cell population like premeiotic germ cells, even slight leakage can undermine the results. The observed germ-cell Ca^{2+} events might be genuine, but the manuscript should clarify how it*

distinguished true germ-cell signals from misexpressed reporters, especially in intravital experiments where anatomical cues are less clear than in slices.“

This is an important point. We acknowledge the reviewer's concern and, therefore, we now describe our criteria for germ cell categorization in the Materials & Methods (p. 24; ll. 22–26) section, which now reads: "*Individual cells were identified by their basal GCaMP6f fluorescence as well as by their position and shape as discerned by tissue autofluorescence. Given some 'leaky' reporter protein expression in offspring from Stra8-Cre x Ai95D mice (see Discussion) we carefully categorized premeiotic germ cells based on (i) their location within the seminiferous epithelium, (ii) their epithelial position close to the basal lamina, and (iii) their spherical shape.*"

Moreover, we added an explanatory statement to the Discussion (p. 18, ll. 17–20) to briefly describe cell identification, refer to Materials & Methods for more details, and also acknowledge the caveat that conservative categorization could have excluded some genuine germ cells from analysis.

Figure R1 shows representative optical sections from two multiphoton *in vivo* imaging experiments, illustrating our approach. Images are acquired at low (a) *versus* high (b) resolution. Tubule walls are indicated by dashed white lines; asterisks indicate blood vessels. Overviews (left) depict merged green (GCaMP6f) and red (autofluorescence) channels, whereas zoom-ins (right) only show basal GCaMP6f fluorescence. The example shown in (b) reveals 'leaky' reporter protein expression in interstitial Leydig cells (open triangle; left). Filled white arrow heads indicate genuine germ cells.

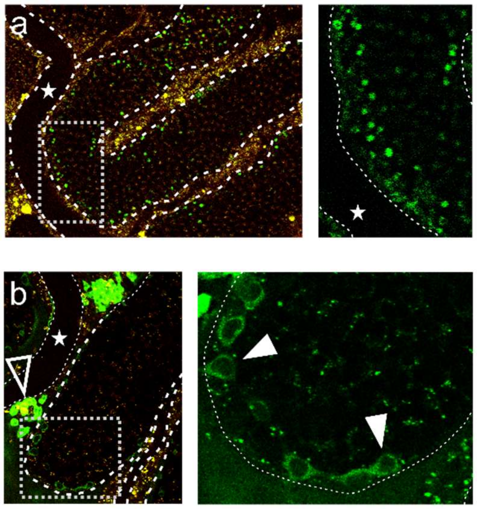

Figure R1: Identification of true germ cells in offspring from Stra8-Cre x Ai95D mice.

To not clutter our manuscript, we suggest not to show Figure R1 (or similar), but rather merely mention our criteria for cell selection within the Materials & Methods and the Discussion sections as outlined above. Should reviewer 1 and/or the editors, however, deem it necessary to include a graphic description, we will be happy to add a corresponding figure to the supplement.

3) *"Interpretation of Germ Cell Ca^{2+} Dynamics: The authors show that germ cells rarely engage in spontaneous Ca^{2+} activity, and when they do, signals are slow and prolonged. This aligns with the biology of intercellular bridges and the comparatively quiescent behavior of type A/B spermatogonia. Still, the conclusion that these cells contribute minimally to Ca^{2+} dynamics of the tubule seems premature. Suppose low-frequency, high-impact Ca^{2+} events exist, perhaps tied to mitotic entry or commitment to meiosis. In that case, such signals might be easily missed at the authors' imaging frame rate or recording duration. A note of caution here would improve the narrative.“*

Again, we thank the reviewer for bringing this up. It is not our intention to dismiss the notion that Ca^{2+} events might play important role(s) in germ cell physiology. In fact, we argue in the Discussion (p. 15, ll. 8–10) that "*Ca^{2+} […] affects the function of thousands of proteins (Clapham, 2007). Accordingly, Ca^{2+} signals exert pleiotropic actions, serving as both positive and negative switches in a plethora of cellular processes (Bootman & Bultynck, 2020).*" Therefore, we would be surprised if, during scenarios such as "*mitotic entry or commitment to*

meiosis", Ca^{2+} signaling would not be involved in some capacity. In the revised manuscript, we now err on the side of caution and make sure that our narrative will not lead to misinterpretation. Relevant new text has been added to the Discussion (p. 15, ll. 14–18), which now reads: "*…such sparse events, when occurring, are prolonged, show slow kinetics, and lack obvious regularity. Note that our findings do not exclude any role(s) of spermatogonial Ca^{2+} signals that act on time scales beyond our measurement window and/or temporal resolution. Profound but slow physiological events, such as mitotic entry or commitment to meiosis, might be characterized by unique Ca^{2+} signatures that evade our present imaging protocol.*"

For clarification, recordings of germ cell Ca^{2+} dynamics were performed at 2 Hz acquisition frequency, both *in vitro* (slices) and *in vivo*. In slices, we recorded for 10 min (9 animals, 24 tubules), with up to four immediate repetitions. *In vivo*, we recorded for 15 min (5 animals, 76 tubules), with up to six interrupted repetitions per field-of-view. All this information is now also available in supporting Table 1 (see above (1)).

4) *"Sertoli Cell Compartmentalization and Signal Diversity: The authors report that brief Ca^{2+} transients are limited to adluminal compartments, while persistent increases occur throughout the entire cell. This is one of the most striking observations in the manuscript, especially in vivo. Their interpretation, that the BTB creates two functionally distinct biochemical regions, seems reasonable. However, interpretations specific to compartments should be made carefully, considering potential optical biases. The basal regions of Sertoli cells in vivo often lie below vascularized interstitial tissue, where light scattering can decrease signal strength. A simple acknowledgment of this possibility would help temper the conclusion."*

Reviewer 1 addresses an important point. Indeed, testicular interstitial vasculature often nestles up against the tubule walls. In an optical section, this can – and sometimes does – obscure fluorescence measurements from basal Sertoli cell regions. We took this into account and, therefore, we routinely recorded 3D stacks of a given field-of-view's vasculature before running functional time-lapse imaging experiments (Figure R2). In case optical access to basal regions of specific tubules was impaired by blood vessels, this area was excluded from analysis. Moreover, we usually confirmed an appropriate signal-to-noise ratio from baseline fluorescence recordings within basal regions. Note also that persistent Ca^{2+} signals, which are not spatially confined and thus span the entire Sertoli cell cytoplasm, are readily detected.

Figure R2 shows a representative example. Here, a two-channel composite image depicts interstitial blood vessels (red; Fig. $R2a_{I}$) that usually cling to the outer surface of tubule walls. The green channel shows GCaMP6f fluorescence (green; Fig. $R2a_{II}$; AMH-Cre x Ai95D mouse). When zooming into an area of the optical section (dashed rectangle in merged Fig. $R2a_{III}$) where a tubule is 'cut' transversally – visualizing both the lumen and entire Sertoli cells with their basal and adluminal segments (Fig. R2b) – optical access to the basal region becomes readily apparent (Fig. $R2b_{II}$). Whenever we compared occurrence of apical *versus* basal transient Ca^{2+} signals, we only selected optical sections that allowed making this distinction. Again, to not clutter our manuscript, we suggest not to show Figure R2, but rather merely mention our selection criteria (see below). Should reviewer 1 and/or the editors, however, deem it necessary to include a graphic description, we will be happy to add a corresponding figure to the supplement.

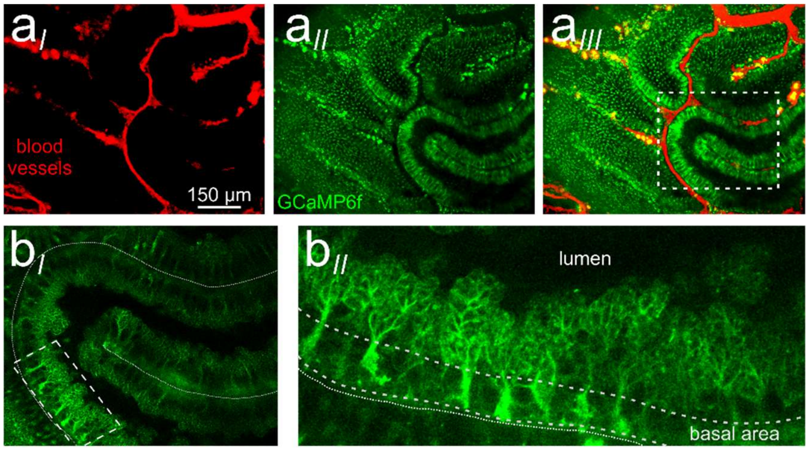

Figure R2: Sertoli Cell compartmentalization in offspring from AMH-Cre x Ai95D mice. (**a**) Overview of a representative field-of-view recorded *in vivo*. Blood vessels and other optical obstructions were manually segmented from z-stacks using LABKIT (**a***I*). The corresponding GCaMP6f fluorescence image demonstrates that vasculature indeed can obscure optical access (**a***II*). The intimate association of tubules and blood vessels becomes apparent in the merged image (**a***III*). For analysis of spatial signal confinement, we therefore chose areas devoid of blood vessel interference (**b**).

That said, reviewer 1 is nonetheless right to suggest that we should acknowledge "*this possibility*." In the revised manuscript, we therefore added a corresponding statement to the Discussion (p. 18, ll. 24–26), which reads: "*Third, while we carefully selected those transversal optical sections during in vivo recordings that allowed imaging of entire Sertoli cells, we cannot rule out that interstitial vasculature could sometimes have decreased fluorescence signal strength during measurements from basal Sertoli cell regions*."

Comments to referee 2:

Reviewer 2 concluded that we "*investigate the role of Ca^{2+} signatures during spermatogenesis in three different cell types of the seminiferous epithelia.*" They pointed out that "*as this is a highly understudied topic, the results are highly relevant and represent a solid characterization upon which further functional studies can orient themselves.*" Notably, reviewer 2 added that we "*nicely dissect the contribution of the different cell types and also by expressing GCaMP6f under the control of cell type specific promoters and confirm the functional data observed in slice recordings in intravital imaging.*" The review was very positive, and reviewer 2 concluded: "*Overall, it is a very well conducted study.*"

Nevertheless, this reviewer provided a "*general comment*" and added that "*several points need further clarification.*"

We thank the reviewer for their thoughtful and instructive comments. All points are valid and, in the revised manuscript, we have addressed them as outlined below:

General comment:

1) *"The discussion would profit from a careful review of the literature about what is known regarding male fertility of IP3R, SERCA or STIM/ORAI knock-out animals."*

We thank the reviewer for bringing this up. We do agree that, especially given our novel results obtained to address this reviewer's specific point 2 (see below), a discussion of fertility-related phenotypes in (male) "*IP3R, SERCA or STIM/ORAI knock-out animals*" is appropriate. Accordingly, we added a paragraph reviewing the available literature to the Discussion (p. 16, ll. 10–18), which now reads: "*In Sertoli cells, the molecular machinery associated with ER generated Ca^{2+} fluxes – including IP3R1, SERCA2, STIM1, and ORAI1 – is present in structures directly related to junction remodeling and spermiation such as TBCs (Adams & Vogl, 2020; Lyon et al., 2017). While knockout of IP3R2 or IP3R3 alone does not affect spermatogenesis (Zhang et al., 2020), IP3R1 knockdown has been shown to alter Sertoli cell actin dynamics, preventing maturation of TBC bulbs (Adams & Vogl, 2020). Homozygous knockout of SERCA2, the major ER Ca^{2+}-ATPase in non-muscle tissues (Prasad et al., 2004), causes embryolethality (Periasamy et al., 1999). While mice lacking expression of both STIM1 and ORAI1 die early postnatally (Feske, 2009), mice solely deficient for ORAI1 survive, though male Orai1$^{-/-}$ mice are sterile and display prominent defects in late-stage elongated spermatid development (Davis et al., 2016).*"

Specific Points:

1) *"Fig. 1: Can the authors explain in more detail the quantification of the Ca^{2+} signals as [au]? Why not given as deltaF/F0? How comparable are the peak of the responses shown in c?"*

To better compare the peak size, signal-to-noise, etc., we now present the original traces shown in Fig. 1C on a unified $\Delta F/F_0$ scale.

2) *"Data in Figure 2f shows that although there is a significant reduction in the number of active cells with 5ppase or IPR3R1KD, there still are 60-70% remaining active cells, thus the statement page 4 bottom "our data suggest that, to a large extent, spontaneous Ca^{2+} signals in the mouse seminiferous tubule are mediated by IP3-dependent Ca^{2+} release from endoplasmic stores", represents an overinterpretation of the data. Did the authors try to*

combine IP3R KD with 5ppase injection? Reducing extracellular Ca^{2+} (Fig. 2b) would disable any type of Ca^{2+} ion channel influx and prolonged Tg treatment (pretreatment for how long?) could cause ER stress to affect other signaling pathways as well. Did the authors try a reversible SERCA inhibitor such as BHQ? Have the authors tried the Orai1 blocker CM5480?“

The reviewer makes several important points. We acknowledge their arguments and, accordingly, we have performed a number of new experiments and now include novel results as well as additional data analysis in the revised manuscript. These new data are now shown as novel Figs. 2C–G as well as 2J&L and outlined below:

(a) *“Data in Figure 2f shows that although there is a significant reduction in the number of active cells with 5ppase or IPR3R1KD, there still are 60-70% remaining active cells, thus the statement page 4 bottom "our data suggest that, to a large extent, spontaneous Ca^{2+} signals in the mouse seminiferous tubule are mediated by IP3-dependent Ca^{2+} release from endoplasmic stores", represents an overinterpretation of the data.*

The reviewer is correct that the majority of cells remain some activity after 5ppase expression or IPR3R1 knockdown. This remaining activity, however, is strongly diminished compared both to control conditions (sham and scrambled RNA injections) and to non-transfected cells from the same samples. Quantitative activity analysis demonstrates a significant reduction in event frequency upon interfering with IP_3 signaling. In the revised manuscript, this new data is shown as Fig. 2J&L. The corresponding Results text (p. 6, ll. 27–29) and figure caption (p. 6, ll. 5–12) have been revised accordingly. Note that additional new data (see below (b-d)) further strengthen our conclusion that “*IP3-dependent Ca^{2+} release from endoplasmic stores*” plays an essential role.

(b) *Reducing extracellular Ca^{2+} (Fig. 2b) would disable any type of Ca^{2+} ion channel influx and prolonged Tg treatment (pretreatment for how long?) could cause ER stress to affect other signaling pathways as well.*

Again, these are important points. We have now performed additional experiments to evaluate whether the duration of exposure to reduced extracellular Ca^{2+} $[Ca^{2+}]_{ex}$ may affect the degree of cellular activity. Data now shown in Fig. 2D demonstrate (*i*) that brief 3 min exposure to low $[Ca^{2+}]_{ex}$ substantially diminishes general activity, and (*ii*) that the percentage of active cells further declines with prolonged exposure. The latter point indicates that, in addition to acute effects mediated by unknown type(s) of plasma membrane Ca^{2+} channels, sustained lowering of $[Ca^{2+}]_{ex}$ mimics the effects exerted by the (ir)reversible SERCA inhibitors thapsigargin and cyclopiazonic-acid, respectively (see (c) below). These new results are now added to Fig. 2D. The corresponding Results text (p. 5, ll. 9–11) and figure caption (p. 5, ll. 26–28) have been revised accordingly.

In additional experiments, using the genetically encoded and thus cell type-specific Ca^{2+} indicator GCaMP6f, we now show that brief 3-min exposure to reduced extracellular Ca^{2+} strongly diminished the number of active TPCs, whereas Sertoli cells were yet to be affected. This immediate impact of Ca^{2+} gradient reversal on TPC signaling indicates a dependence on extracellular Ca^{2+} influx on a per-event basis, whereas Sertoli cells can maintain Ca^{2+} signaling on ER-based mechanisms for longer periods. Notably, these findings are in line with the notion of use-dependent SOCE exhaustion upon ORAI inhibition in Sertoli cells (see below (d)). We now show these data in new supplementary Fig S1, and we added corresponding text to the Results (p. 8, ll. 6–9) and Discussion (p. 16, ll. 2–6).

Thapsigargin pretreatment lasted for 30–40 min, depending on the specific time course of Ca^{2+} store depletion (as assessed by monitoring intracellular Ca^{2+} levels during treatment at low

image acquisition rate (0.2 Hz)). Details have been added to the corresponding Materials & Methods text (p. 21, ll. 7–15).

(c) *Did the authors try a reversible SERCA inhibitor such as BHQ?*

By investigating effects of the reversible SERCA inhibitor cyclopiazonic-acid (CPA), we have now included additional evidence supporting a role of Ca^{2+} release from the endoplasmic reticulum. BHQ and CPA both inhibit SERCA by occupying the Ca^{2+} access channel delimited by transmembrane segments M1–M4 (Chen et al., 2020). As for experiments testing thapsigargin (see (b) above), CPA pretreatment duration (<30 min) depended the observation of a slow rise in intracellular Ca^{2+} and subsequent recovery. Representative traces from an original recording and the corresponding data quantification have been added to Fig. 2C&D. The Results text (p. 5, ll. 11–15) and figure caption (pp. 5, ll. 25–28) have been revised accordingly.

(d) *Have the authors tried the Orai1 blocker CM5480?*

We thank the reviewer for suggesting this highly informative experiment. Following the reviewer's advice, we initially attempted to procure CM5480. As this drug is not commercially available, we contacted the manufacturer CalciMedica (https://calcimedica.com/) as well as academic researchers who recently published studies in which CM5480 was used. Both Dr. József Maléth (University of Szeged, Hungary) and Dr. Fabrice Antigny (INSERM, France) confirmed that CM5480 can only be obtained from the company directly. Unfortunately, our emails to Ken Stauderman, the Chief Scientific Officer of CalciMedica, remained unanswered. However, Dr. Maléth wrote: "*Alternatively, and this may be more suitable for a revision, you can also use GSK-7975A. This is another selective Orai1 inhibitor, which is commercially available. In our systems it had similar channel blocking effectiveness (actually the inhibition kinetic was somewhat faster)*." Accordingly, we performed a series of new experiments investigating effects upon inhibition of Orai1 by GSK-7975A. The results of these recordings demonstrate that, compared to control conditions, exposure to GSK-7975A (10 µM; 10 min) substantially reduced the number of active cells per tubule area. This effect became evident for both putative TPCs and Sertoli cells, as categorized by their distinct shape and location. Moreover, cells that maintained residual activity in presence of the drug displayed fewer Ca^{2+} transients. Together, these results suggest that ORAI-mediated SOCE is required to maintain spontaneous Ca^{2+} signaling integrity, likely acting in a use-dependent manner. We now show these data as new Fig. 2E–G. Accordingly, we have revised the Results text (p. 6, ll. 14–23), figure caption (pp. 5–6, ll. 28–1), and Discussion (p. 16, ll. 7–21).

We note that we were able to perform these new experiments (as well as new slice experiments referred to in (4) and (6) below) with more recently purchased confocal microscopes. Therefore, we generally acquired larger data sets with improved resolution and signal-to-noise ratio. This also allowed us, in close collaboration with our colleagues Dr. Johannes Stegmaier (Heinrich Heine University Düsseldorf) and Melissa Franke, M.Sc., to analyze our additional recordings using novel and improved image registration algorithms and analysis code. The procedures are described in Materials & Methods (p. 26, ll. 6–15; p. 27, ll. 10–14) and the corresponding custom Python code will be available in a repository (www.XXX.XX) upon publication. Accordingly, Dr. Stegmaier and Ms. Franke have been added to the list of authors.

Together, several new lines of evidence – i.e., quantitative analysis of residual activity after 5ppase expression or IPR3R1 knockdown (Fig. 2J&L), progressive decline of cell activity with prolonged exposure to reduced $[Ca^{2+}]_{ex}$ (Fig. 2D), similar results obtained upon SERCA

inhibition with either thapsigargin or CPA, respectively (Fig. 2C&D), as well as evidence for a critical, though cell type-specific role of store-operated Ca^{2+} entry (Fig. 2E–G) – strongly suggest that seminiferous tubule Ca^{2+} signaling, particularly in Sertoli cells, is largely driven by mechanisms controlling Ca^{2+} release from the ER. Extending previous work (Adams & Vogl, 2020), we propose that IP3R1, SERCA2, and ORAI1 orchestrate these processes.

3) "Fig. 3: the example traces shown in f_{III} for the Sertoli cells do not represent the majority of cells that show a similar degree of frequent oscillations as quantified in g,h."

The reviewer is right. In our revised manuscript, we now show two example traces for germ cells (D_{III}), TPCs (E_{III}), and Sertoli cells (F_{III}), respectively. These traces were chosen to represent each of the observed phenotypes, i.e., sporadically active *versus* quiescent (D_{III}), frequent *versus* sporadic (E_{III}), as well as sporadic *versus* frequent (F_{III}), respectively. Colors correspond to data "*quantified in g,h.*"

4) "The observation that spontaneous Sertoli cells activity occurs in hot spots is interesting. Do these hotspots correlate with the number of Spermatid nuclei? Are they correlated with tubulobulbar complexes? Are these also seen in slice recordings and can nuclei be visualized simultaneously?"

We embrace the reviewer's suggestion and, accordingly, we have performed a new series of experiments to address these questions. While we are not able to investigate potential correlation between hot spot occurrence and "*the number of Spermatid nuclei*" *in vivo*, we performed additional slice recordings combined with *post-hoc* visualization of nuclei. These new results are now depicted as Fig. 5E–K. The Results text (pp. 10–11, ll. 36–6), figure caption (p. 10, ll. 20–34), Discussion (p. 16, ll. 22–27), and Materials & Methods (pp. 22–23, ll. 30–2; pp. 25, ll. 14–31) have been revised accordingly.

Post-hoc nuclear staining / imaging in slices enables categorization of recorded tubules into three groups: putative early (I–V), middle (VI–VIII), and late (IX–XII) spermatogenic stages (Hess & De Franca, 2008). With spermiation occurring at stage VIII (see Fig. 1A), the late stages IX to XII are characterized by absence of late spermatids from the epithelium (Adams & Vogl, 2020). Immediately prior to spermiation (i.e., during middle stages VI–VIII), apical tubulobulbar complexes (TBCs) are formed in preparation of sperm release and late elongated spermatids are densely packed in characteristic adluminal clusters (Vogl et al., 2013). In the early stages I–V, elongated spermatids are less condensed and scattered more loosely within the apical seminiferous epithelium (Hess & De Franca, 2008). The above anatomical characteristics of both middle and late stages – i.e., clustered and highly condensed late spermatids (middle) *versus* absence of late spermatids (late) – can be inferred from nuclear staining. Example images for this categorization are now shown as Fig. 5G. We defined hot spot activity in slices according to a multimodal distribution of active Sertoli cells per area, with regions containing > 150 active cells / mm^2 being considered a hot spot. To err on the side of caution, we did not attempt to label early stage (I–V) tubules, since these are more diffuse and thus hard to unequivocally identify.

When plotting activity density / hot spots as a function of middle *versus* late spermatogenic cycle stage (new Fig. 5I), we found that hot spots are essentially confined to middle stages prior to spermiation. Moreover, in middle stage tubules, Ca^{2+} signals display slower decay and, thus, they last significantly longer than the more transient signals observed during late stages new Fig. 5J&K). Our finding that hot spot activity correlates with spermatogenic cycle stage

supports the notion that local ER-mediated Ca^{2+} fluctuations in Sertoli cells may exert mechanistic control over events related to TBC formation and spermiation (Lyon et al., 2017b).

5) "Fig. 6: please exchange colors as violet ROI boundaries in aIII are very difficult to see. Also, what is the reason for the completely different time scale of Ca^{2+} signals shown in e_I (2 min) compared to d_I (30s)?"

We followed the reviewer's advice and changed the colors of "*ROI boundaries*" throughout subpanels A_I – A_{IV} to improve readability.

The reason to show the representative traces in C_I – E_I on different time scales is that, as compared to C_I and D_I, the recordings shown in E_I depict a qualitatively different phenomenon. While traces in C_I and D_I demonstrate relatively frequent transient activity where individual signals typically last few seconds, persistent activity is shown in E_I. The latter usually lasts several minutes. Showing both activity patterns on the same time scale would render it difficult to discern at least one of them.

6) "Do differences in Ca^{2+} signatures in Sertoli cells during FSH treatment (see Fig 7g) correlate with spermiation? Have the authors investigated this in immature male mice? Is there an age dependency of the effect?"

The reviewer makes several excellent points, which prompted us to perform additional *in vivo* experiments in juvenile/pubescent (postnatal days 26–28) male animals. These experiments allowed us to determine (*i*) whether Ca^{2+} signaling signatures vary with age under control conditions, and (*ii*) if FSH treatment effects are age-dependent. The results of these experiments are now depicted as Figs. S2 & S4. The Results text (p. 10, ll. 8–9; p. 13, ll. 10–16), figure captions (p. 30, ll. 10–20; pp. 30–31, ll. 37–8), and Discussion (p. 18, ll. 6–12) have been revised accordingly.

Briefly, we analyzed both transient and persistent Sertoli cell activity, tested whether signals are spatially confined and occur in dynamic hot spots, and we investigated if Sertoli cell *in vivo* signaling in juvenile mice is affected by FSH. When comparing Sertoli cell Ca^{2+} dynamics and their susceptibility to FSH in adult *versus* juvenile males, we note that patterns of transient and spatially restricted activity (i.e. hot spots and adluminal confinement) are very similar. By contrast, persistent and spatially uniform Ca^{2+} elevations, while occurring somewhat less frequently in juveniles, remain unaltered by FSH. While this intriguing discrepancy will have to be addressed in future studies, our observation substantiates that transient and persistent Ca^{2+} signals are controlled by different mechanisms, mediate different physiological processes, and are governed by different endocrine programs.

Comments to the Academic Editor:

The Academic Editor “*has concerns regarding the gonadotropin treatment experiments and conclusions, and that this should be addressed experimentally to support the claims on LH and FSH treatment. These hormones were given to non-castrated males, so the levels would have been supraphysiological as FSH affects Sertoli cells in the seminiferous tubules and LH affects testosterone production by Leydig cells located in the interstitium between tubules. Whether there is some direct effect of the gonadotropins on the cells exhibiting calcium signaling, or if there is some indirect effect through suppression of the hypothalamic-pituitary-testis axis was not explored, and it is not mentioned how much of the hormones was given and at what time relative to the imaging. Thus, additional experiments are needed to support the claim that these gonadotropins regulate Ca^{2+} signals, particularly at physiological levels.*”

We appreciate the Academic Editor’s comments. Below, we respond in detail to their concerns, we outline the respective rationale behind our experimental strategies, we describe “*additional experiments*” we performed to support our claims, and we refer to the relevant sections in the revised manuscript. The concerns raised by the Academic Editor are addressed individually:

1) *“Hormones were given to non-castrated males, so the levels would have been supraphysiological as FSH affects Sertoli cells in the seminiferous tubules and LH affects testosterone production by Leydig cells located in the interstitium between tubules. […] It is not mentioned how much of the hormones was given and at what time relative to the imaging.”*

We thank the Academic Editor for bringing this up. While we are aware that using “*non-castrated males*” prevents experimental control of systemic gonadotropin levels, we opted for this strategy to avoid pitfalls inherent to castration models. Obviously, surgical or bloodless castration could not be performed. Functional castration (i.e., chemical castration or GnRH immunocastration) causes rapid degeneration of the seminiferous epithelium, collapse of tubule structure, Leydig cell regress, Sertoli cell entry into a quiescent state, and loss of blood–testis barrier integrity (C. Wang et al., 2023). Consequently, circulating testosterone levels drop dramatically whereas gonadotropins increase from the loss of negative inhibin B feedback.

The Academic Editor makes an important point as we simply forgot to provide information on the dose and timing of hormone administration. We sincerely apologize for this oversight. During *in vivo* experiments, we injected a total volume of 50 µl i.p. (25 µl gonadotropin solution; 25 µl saline). In male C57BL/6 mice, reports on mean serum FSH levels range between 10 ng/ml and 75 ng/ml (33 ± 26 ng/ml; mean ± SD (Jeong et al., 2009; Ma et al., 2022; Puett et al., 2005; Schneider et al., 2005; Singh & Handelsman, 1996)). FSH concentrations are, however, an order of magnitude higher than basal LH under intact negative feedback. Notably, LH release is pulsatile (~0.3–0.7 pulses/hour (Steyn et al., 2013)) with an LH half-life of ~12 min, so LH levels are inherently variable. To mimic a gonadotropin surge in an adult mouse (~30 g) with a total blood volume of ~2 ml, we thus injected 501 ng FSH and 90 ng LH, respectively, leading to calculated initial serum levels of 250.5 ng/ml (FSH) or 45 ng/ml (LH). Notably, this is far less than the amounts reported by others for similar experiments (0.4–15 µg/ml (Abel et al., 2009; O’Shaughnessy et al., 1992, 2010; H. Wang et al., 2016)). We have now added this information to Materials & Methods (p. 21, ll. 25–29).

Following a standard experimental protocol, multiphoton imaging data from a given field-of-view was acquired as 15 min sequences at ~1 h intervals (mean interval 58.4 ± 16.6 min). After control recordings under physiological conditions, we injected either gonadotropin and resumed imaging after 31.4 ± 9.7 min (FSH) or 23.3 ± 9.9 min (LH), respectively. This information has also been added to Materials & Methods (p. 24, ll. 27–31).

2) *“Whether there is some direct effect of the gonadotropins on the cells exhibiting calcium signaling, or if there is some indirect effect through suppression of the hypothalamic-pituitary-testis axis was not explored. […] Thus, additional experiments are needed to support the claim that these gonadotropins regulate Ca^{2+} signals, particularly at physiological levels.”*

We thank the Academic Editor for raising this important point. To explore whether gonadotropins affect Sertoli cell Ca^{2+} signaling directly or if the effects observed result from “*suppression of the hypothalamic-pituitary-testis axis*”, *in vivo* experiments are not suitable (see above (1)). Therefore, we decided to test whether physiological FSH levels directly affect Sertoli cell Ca^{2+} signaling in isolated seminiferous tubules / slices. We focused on FSH because of its more dramatic effects on Ca^{2+} mobilization and, simply, because the isolated tubule preparation lacks Leydig cells which are the testicular target of LH. We recorded Ca^{2+} signals in putative Sertoli cells during 30 min exposure to FSH (33 ng/ml; corresponding to physiological mean serum FSH levels reported by others (see above)). While we are aware that the most striking effects exerted by FSH treatment *in vivo* (i.e., loss of both hot spot and persistent activity) gradually develop over prolonged periods of time (up to 3 h post injection), recordings in isolated tubules only lasted ≤ 1 h. For this duration, control recordings revealed stable Ca^{2+} signaling phenotypes. Notably, even within only 30 min of FSH exposure, gonadotropin treatment strongly affected Sertoli cell Ca^{2+} signals. In presence of FSH, Ca^{2+} transients displayed substantially faster decay, leading to much shorter individual events. These results demonstrate that FSH directly regulates “*Ca^{2+} signals, particularly at physiological levels.*” Our new results are now presented as Fig. 8H–L. We added a corresponding new paragraph to the Discussion (pp. 17–18, ll. 31–5), which now reads: “*In vitro experiments allow us to monitor whether physiological serum concentrations of FSH directly affect seminiferous tubule Ca^{2+} signaling. In adult male C57BL/6 mice, serum FSH levels between 10 ng/ml and 75 ng/ml (33 ± 26 ng/ml; mean ± SD) have been reported (Jeong et al., 2009; Ma et al., 2022; Puett et al., 2005; Schneider et al., 2005; Singh & Handelsman, 1996). We therefore challenged isolated tubules with 33 ng/ml FSH. While in vitro experiments allow precise control of hormone concentration, they lack the power of prolonged in vivo observations over several hours. Long-term effects, such as hot spot dynamics and their endocrine control, thus evade in vitro observation. Nonetheless, physiological FSH concentrations directly affect spontaneous Sertoli cell Ca^{2+} signal kinetics in isolated tubules, strongly reducing their duration and thus likely their impact*.” The corresponding Results text (p. 14, ll. 5–19) and figure caption (pp. 13–14, ll. 32–3) have been revised accordingly.

Completed formatting changes:

1) *"We noticed that you used "data not shown" in the manuscript. We do not allow these references, as the PLOS data access policy requires that all data be either published with the manuscript or made available in a publicly accessible database. Please amend the supplementary material to include the referenced data or remove the references."*

We removed the references.

2) *"We do not publish any copyright or trademark symbols that usually accompany proprietary names, eg (R), (C), or TM (e.g. next to drug or reagent names). Please remove all instances of trademark/copyright symbols throughout the text, including ™ on page 17."*

We removed all instances of trademark/copyright symbols throughout the text.

3) *"We ask that a manuscript source file is provided at Revision. Please upload your manuscript file as a .doc, .docx, .rtf or .tex."*

done

4) *"Please upload the main figures as separate figure files in .tif or .eps format."*

done

5) *"Some material included in your submission may be copyrighted. According to PLOS's copyright policy, authors who use figures or other material (e.g., graphics, clipart, maps) from another author or copyright holder must demonstrate or obtain permission to publish this material under the Creative Commons Attribution 4.0 International (CC BY 4.0) License used by PLOS journals. Please closely review the details of PLOS's copyright requirements here: PLOS Licenses and Copyright. If you need to request permissions from a copyright holder, you may use PLOS's Copyright Content Permission form.*

Please respond directly to this email and provide any known details concerning your material's license terms and permissions required for reuse, even if you have not yet obtained copyright permissions or are unsure of your material's copyright compatibility. Once you have responded and addressed all other outstanding technical requirements, you may resubmit your manuscript within Editorial Manager.

Potential Copyright Issues:

-Figures 1a,2a,2e,3,4,and 7a. Please confirm whether you drew the images / clip-art within the figure panels by hand. If you did not draw the images, please provide (a) a link to the source of the images or icons and their license / terms of use; or (b) written permission from the copyright holder to publish the images or icons under our CC BY 4.0 license. Alternatively, you may replace the images with open source alternatives."

We confirm that all images / clip-art has been drawn by us. No material included in our submission is copyrighted.
