# Supplementary material for: Cell type-specific Ca2+ signals govern mouse seminiferous tubule physiology
Source: PLoS Biol. 2026 Jul 24;24(7):e3003910. doi: 10.1371/journal.pbio.3003910 (PMC13399359; doi:10.1371/journal.pbio.3003910)
Supplement: Peer review history

# Editor report: Decision Letter 0

**DOI:** 10.1371/journal.pbio.3003910.r001

**Authors:** Ines Alvarez-Garcia

23 Sep 2025

Dear Dr Spehr,

Thank you for submitting your manuscript entitled "Cell type-specific Ca2+ signals govern mouse seminiferous tubule physiology" for consideration as a Research Article by PLOS Biology.

Your manuscript has now been evaluated by the PLOS Biology editorial staff as well as by an academic editor with relevant expertise and I am writing to let you know that we would like to send your submission out for external peer review.

However, before we can send your manuscript to reviewers, we need you to complete your submission by providing the metadata that is required for full assessment. To this end, please login to Editorial Manager where you will find the paper in the 'Submissions Needing Revisions' folder on your homepage. Please click 'Revise Submission' from the Action Links and complete all additional questions in the submission questionnaire.

Once your full submission is complete, your paper will undergo a series of checks in preparation for peer review. After your manuscript has passed the checks it will be sent out for review. To provide the metadata for your submission, please Login to Editorial Manager (https://www.editorialmanager.com/pbiology) within two working days, i.e. by Sep 25 2025 11:59PM.

If your manuscript has been previously peer-reviewed at another journal, PLOS Biology is willing to work with those reviews in order to avoid re-starting the process. Submission of the previous reviews is entirely optional and our ability to use them effectively will depend on the willingness of the previous journal to confirm the content of the reports and share the reviewer identities. Please note that we reserve the right to invite additional reviewers if we consider that additional/independent reviewers are needed, although we aim to avoid this as far as possible. In our experience, working with previous reviews does save time.

If you would like us to consider previous reviewer reports, please edit your cover letter to let us know and include the name of the journal where the work was previously considered and the manuscript ID it was given. In addition, please upload a response to the reviews as a 'Prior Peer Review' file type, which should include the reports in full and a point-by-point reply detailing how you have or plan to address the reviewers' concerns.

During the process of completing your manuscript submission, you will be invited to opt-in to posting your pre-review manuscript as a bioRxiv preprint. Visit http://journals.plos.org/plosbiology/s/preprints for full details. If you consent to posting your current manuscript as a preprint, please upload a single Preprint PDF.

Feel free to email us at plosbiology@plos.org if you have any queries relating to your submission.

Kind regards,

Ines

--

Ines Alvarez-Garcia, PhD

Senior Editor

PLOS Biology

ialvarez-garcia@plos.org

# Peer review: Decision Letter 1

**DOI:** 10.1371/journal.pbio.3003910.r002

**Authors:** Ines Alvarez-Garcia

17 Dec 2025

Dear Dr Spehr,

Thank you for your patience while your manuscript entitled "Cell type-specific Ca2+ signals govern mouse seminiferous tubule physiology" was peer-reviewed at PLOS Biology, and please accept again my sincere apologies for the delay in sending you our decision. The manuscript has now been evaluated by the PLOS Biology editors, an Academic Editor with relevant expertise, and by two independent reviewers.

The reviews are attached below. As you will see, the reviewers find the conclusions interesting, but they also raise several issues that would need to be addressed before we can consider the manuscript for publication. Reviewer 1 raises several points, including the statistic details on sampling, which need to be clearly explained, and the cellular identification and driver line specificity, which seems to be leaky. This reviewer also thinks that it should be explained how true germ-cell signals are discriminated from misexpressed reporters and that caution should be used for the interpretation of the germ cell calcium dynamics and the specificity of Ca2+ signals to some compartments. Reviewer 2 mostly asks for several clarifications, including how the Ca2+ signals are quantified.

In light of the reviews and after consulting with the Academic Editor and the rest of the team, we would like to invite you to revise the work to thoroughly address the reviewers' reports. Given the extent of revision needed, we cannot make a decision about publication until we have seen the revised manuscript and your response to the reviewers' comments. Your revised manuscript is likely to be sent for further evaluation by all or a subset of the reviewers.

In addition to these revisions, you will need to complete some formatting changes, which you will receive in a follow up email. A member of our team will be in touch with a set of requests shortly.

We expect to receive your revised manuscript within 3 months. Please email us (plosbiology@plos.org) if you have any questions or concerns, or would like to request an extension.

At this stage, your manuscript remains formally under active consideration at our journal; please notify us by email if you do not intend to submit a revision so that we may withdraw it.

**IMPORTANT - SUBMITTING YOUR REVISION**

Your revisions should address the specific points made by each reviewer. Please submit the following files along with your revised manuscript:

1. A 'Response to Reviewers' file - this should detail your responses to the editorial requests, present a point-by-point response to all of the reviewers' comments, and indicate the changes made to the manuscript.

*NOTE: In your point-by-point response to the reviewers, please provide the full context of each review. Do not selectively quote paragraphs or sentences to reply to. The entire set of reviewer comments should be present in full and each specific point should be responded to individually, point by point.

You should also cite any additional relevant literature that has been published since the original submission and mention any additional citations in your response.

2. In addition to a clean copy of the manuscript, please also upload a 'track-changes' version of your manuscript that specifies the edits made. This should be uploaded as a "Revised Article with Changes Highlighted" file type.

3. Resubmission Checklist

When you are ready to resubmit your revised manuscript, please refer to this resubmission checklist: https://plos.io/Biology_Checklist

To submit a revised version of your manuscript, please go to https://www.editorialmanager.com/pbiology/ and log in as an Author. Click the link labelled 'Submissions Needing Revision' where you will find your submission record.

Please make sure to read the following important policies and guidelines while preparing your revision and fulfil the editorial requests:

a) *PLOS Data Policy*

Please note that as a condition of publication PLOS' data policy (http://journals.plos.org/plosbiology/s/data-availability) requires that you make available all data used to draw the conclusions arrived at in your manuscript. If you have not already done so, you must include any data used in your manuscript either in appropriate repositories, within the body of the manuscript, or as supporting information (N.B. this includes any numerical values that were used to generate graphs, histograms etc.). Please also indicate in each figure legend where the data can be found. For an example see here: http://www.plosbiology.org/article/info%3Adoi%2F10.1371%2Fjournal.pbio.1001908#s5

b) *Published Peer Review*

Please note while forming your response, if your article is accepted, you may have the opportunity to make the peer review history publicly available. The record will include editor decision letters (with reviews) and your responses to reviewer comments. If eligible, we will contact you to opt in or out. Please see here for more details:

https://blogs.plos.org/plos/2019/05/plos-journals-now-open-for-published-peer-review/

c) *Blot and Gel Data Policy*

Please provide the original, uncropped and minimally adjusted images supporting all blot and gel results reported in an article's figures or Supporting Information files. We will require these files before a manuscript can be accepted so please prepare them now, if you have not already uploaded them. Please carefully read our guidelines for how to prepare and upload this data: https://journals.plos.org/plosbiology/s/figures#loc-blot-and-gel-reporting-requirements

d) *Blurb*

Please also provide a blurb which (if accepted) will be included in our weekly and monthly Electronic Table of Contents, sent out to readers of PLOS Biology, and may be used to promote your article in social media. The blurb should be about 30-40 words long and is subject to editorial changes. It should, without exaggeration, entice people to read your manuscript. It should not be redundant with the title and should not contain acronyms or abbreviations. For examples, view our author guidelines: https://journals.plos.org/plosbiology/s/revising-your-manuscript#loc-blurb

e) *Protocols deposition*

To enhance the reproducibility of your results, we recommend that if applicable you deposit your laboratory protocols in protocols.io, where a protocol can be assigned its own identifier (DOI) such that it can be cited independently in the future. For instructions see: https://journals.plos.org/plosbiology/s/submission-guidelines#loc-materials-and-methods

Thank you again for your submission to our journal. We hope that our editorial process has been constructive thus far, and we welcome your feedback at any time. Please don't hesitate to contact us if you have any questions or comments.

Sincerely,

Ines

--

Ines Alvarez-Garcia, PhD

Senior Editor

PLOS Biology

ialvarez-garcia@plos.org

------------------------------------

Reviewers' comments

Rev. 1:

In the manuscript "Cell type-specific Ca2+ signals govern mouse seminiferous tubule physiology," the authors present an ambitious and technically advanced study examining Ca²⁺ signaling across the three main cellular components of the mouse seminiferous tubule (Sertoli cells, peritubular myoid cells, and premeiotic germ cells) using both acute tubule slices and intravital multiphoton imaging. The manuscript heavily depends on Cre-driver specificity to assert cell-type-specific Ca²⁺.

Their objective is to define cell-type-specific Ca²⁺ "fingerprints" and to suggest how these signatures influence aspects of seminiferous tubule physiology, including endocrine responsiveness, addressing a genuine gap in reproductive physiology: how Ca²⁺ signals are organized within the intact seminiferous epithelium and how these signals differ among supporting cell types. responses. Cytosolic Ca2+ levels were measured in three cell types of the mouse seminiferous tubule to better understand the physiological mechanisms regulating mammalian spermatogenesis, which still largely remains a biological black box. Both in vitro and in vivo studies show clear, cell-specific signal patterns that (i) have distinct spatiotemporal signatures, (ii) depend on IP3-mediated Ca2+ release from endoplasmic stores, and (iii) are influenced by gonadotropins.

While the imaging technique is technically proficient and some observations are truly intriguing, the manuscript frequently relies more on interpretation than direct evidence. Many key claims are undermined by methodological constraints, insufficient validation of cell identities, and overambitious claims about what the data can legitimately support. Currently, the manuscript appears to be shaped by the capabilities of the imaging system rather than by considerations of biological importance, causality, or physiological relevance.

Major comments:

Statistical transparency: For a study that relies heavily on event frequency, duration, and oscillatory behavior, clear information about sampling is crucial. Several analyses would benefit from explicitly reporting: the number of tubules versus the number of cells per animal, variability between animals, the number of signals recorded across how many imaging intervals, and the criteria for defining "frequent" versus "sporadic" events. A brief supplemental table could address most of these points.

Cellular Identification and Driver Line Specificity: The manuscript heavily depends on Cre-driver specificity to assert cell-type-specific Ca²⁺ responses. Although AMH-Cre and SMMHC-CreERT2 are generally reliable, the authors acknowledge leakage issues with Stra8-Cre mice. This point is significant and warrants more emphasis. When studying a very sparse cell population like premeiotic germ cells, even slight leakage can undermine the results. The observed germ-cell Ca²⁺ events might be genuine, but the manuscript should clarify how it distinguished true germ-cell signals from misexpressed reporters, especially in intravital experiments where anatomical cues are less clear than in slices.

Interpretation of Germ Cell Ca²⁺ Dynamics: The authors show that germ cells rarely engage in spontaneous Ca²⁺ activity, and when they do, signals are slow and prolonged. This aligns with the biology of intercellular bridges and the comparatively quiescent behavior of type A/B spermatogonia. Still, the conclusion that these cells contribute minimally to Ca²⁺ dynamics of the tubule seems premature. Suppose low-frequency, high-impact Ca²⁺ events exist, perhaps tied to mitotic entry or commitment to meiosis. In that case, such signals might be easily missed at the authors' imaging frame rate or recording duration. A note of caution here would improve the narrative.

Sertoli Cell Compartmentalization and Signal Diversity: The authors report that brief Ca²⁺ transients are limited to adluminal compartments, while persistent increases occur throughout the entire cell. This is one of the most striking observations in the manuscript, especially in vivo. Their interpretation, that the BTB creates two functionally distinct biochemical regions, seems reasonable. However, interpretations specific to compartments should be made carefully, considering potential optical biases. The basal regions of Sertoli cells in vivo often lie below vascularized interstitial tissue, where light scattering can decrease signal strength. A simple acknowledgment of this possibility would help temper the conclusion.

Rev. 2:

Fischoeder et al. investigate the role of Ca2+ signatures during spermatogenesis in three different cell types of the seminiferous epithelia. As this is a highly understudied topic, the results are highly relevant and represent a solid characterization upon which further functional studies can orient themselves. The authors nicely dissect the contribution of the different cell types and also by expressing GCaMP6f under the control of cell type specific promoters and confirm the functional data observed in slice recordings in intravital imaging. Overall, it is a very well conducted study. The discussion would profit from a careful review of the literature about what is known regarding male fertility of IP3R, SERCA or STIM/ORAI knock-out animals.

Several points need further clarification:

1. Fig. 1: Can the authors explain in more detail the quantification of the Ca2+ signals as [au]? Why not given as deltaF/F0? How comparable are the peak of the responses shown in c?

2. Data in Figure 2f shows that although there is a significant reduction in the number of active cells with 5ppase or IPR3R1KD, there still are 60-70% remaining active cells, thus the statement page 4 bottom "our data suggest that, to a large extent, spontaneous Ca2+ signals in the mouse seminiferous tubule are mediated by IP3-dependent Ca2+ release from endoplasmic stores", represents an overinterpretation of the data. Did the authors try to combine IP3R KD with 5ppase injection? Reducing extracellular Ca2+ (Fig. 2b) would disable any type of Ca2+ ion channel influx and prolonged Tg treatment (pretreatment for how long?) could cause ER stress to affect other signaling pathways as well. Did the authors try a reversible SERCA inhibitor such as BHQ? Have the authors tried the Orai1 blocker CM5480?

3. Fig. 3: the example traces shown in fIII for the Sertoli cells do not represent the majority of cells that show a similar degree of frequent oscillations as quantified in g,h.

4. The observation that spontaneous Sertoli cells activity occurs in hot spots is interesting. Do these hotspots correlate with the number of Spermatid nuclei? Are they correlated with tubulobulbar complexes? Are these also seen in slice recordings and can nuclei be visualized simultaneously?

5. Fig. 6: please exchange colors as violet ROI boundaries in aIII are very difficult to see. Also, what is the reason for the completely different time scale of Ca2+ signals shown in eI (2 min) compared to dI (30s)?

6. Do differences in Ca2+ signatures in Sertoli cells during FSH treatment (see Fig 7g) correlate with spermiation? Have the authors investigated this in immature male mice? Is there an age dependency of the effect?

# Author comment: Author response to Decision Letter 2

**DOI:** 10.1371/journal.pbio.3003910.r003

4 May 2026

Attachment Submitted filename: Response to Reviewers.pdf

# Peer review: Decision Letter 2

**DOI:** 10.1371/journal.pbio.3003910.r004

**Authors:** Ines Alvarez-Garcia

22 Jun 2026

Dear Dr Spehr,

Thank you for your patience while we considered your revised manuscript entitled "Cell type-specific Ca2+ signals govern mouse seminiferous tubule physiology" for publication as a Research Article at PLOS Biology. This revised version of your manuscript has been evaluated by the PLOS Biology editors, the Academic Editor and the two original reviewers.

Based on the reviews, we are likely to accept this manuscript for publication, provided you satisfactorily address the data and other policy-related requests stated below my signature.

As you address these items, please take this last chance to review your reference list to ensure that it is complete and correct. If you have cited papers that have been retracted, please include the rationale for doing so in the manuscript text, or remove these references and replace them with relevant current references. Any changes to the reference list should be mentioned in the cover letter that accompanies your revised manuscript.

In addition to these revisions, you may need to complete some formatting changes, which you will receive in a follow up email. A member of our team will be in touch with a set of requests shortly. If you do not receive a separate email within a few days, please assume that checks have been completed, and no additional changes are required.

We expect to receive your revised manuscript within two weeks.

To submit your revision, please go to https://www.editorialmanager.com/pbiology/ and log in as an Author. Click the link labelled 'Submissions Needing Revision' to find your submission record. Your revised submission must include the following:

- a cover letter that should detail your responses to any editorial requests, if applicable, and whether changes have been made to the reference list

- a Response to Reviewers file that provides a detailed response to the reviewers' comments (if applicable, if not applicable please do not delete your existing 'Response to Reviewers' file.)

- a track-changes file indicating any changes that you have made to the manuscript.

NOTE: If Supporting Information files are included with your article, note that these are not copyedited and will be published as they are submitted. Please ensure that these files are legible and of high quality (at least 300 dpi) in an easily accessible file format. For this reason, please be aware that any references listed in an SI file will not be indexed. For more information, see our Supporting Information guidelines:

https://journals.plos.org/plosbiology/s/supporting-information

*Published Peer Review History*

Please note that you may have the opportunity to make the peer review history publicly available. The record will include editor decision letters (with reviews) and your responses to reviewer comments. If eligible, we will contact you to opt in or out. Please see here for more details:

https://plos.org/published-peer-review-history/

*Press*

Should you, your institution's press office or the journal office choose to press release your paper, please ensure you have opted out of Early Article Posting on the submission form. We ask that you notify us as soon as possible if you or your institution is planning to press release the article.

*Protocols deposition*

To enhance the reproducibility of your results, we recommend that if applicable you deposit your laboratory protocols in protocols.io, where a protocol can be assigned its own identifier (DOI) such that it can be cited independently in the future. Additionally, PLOS ONE offers an option for publishing peer-reviewed Lab Protocol articles, which describe protocols hosted on protocols.io. Read more information on sharing protocols at https://plos.org/protocols?utm_medium=editorial-email&utm_source=authorletters&utm_campaign=protocols

Please do not hesitate to contact me should you have any questions.

Sincerely,

Ines

--

Ines Alvarez-Garcia, PhD

Senior Editor

PLOS Biology

ialvarez-garcia@plos.org

------------------------------------------------------------------------

ETHICS STATEMENT:

Thank you for providing the Ethics statement. We noticed that you included an statement in Methods, which is missing the protocol number, and then added another one under 'Additional Information'. Please combine them into one and delete the second statement in Additional Information.

------------------------------------------------------------------------

DATA POLICY:

You may be aware of the PLOS Data Policy, which requires that all data be made available without restriction: http://journals.plos.org/plosbiology/s/data-availability. For more information, please also see this editorial: http://dx.doi.org/10.1371/journal.pbio.1001797

Note that we do not require all raw data. Rather, we ask that all individual quantitative observations that underlie the data summarized in the figures and results of your paper be made available in one of the following forms:

1) Supplementary files (e.g., excel). Please ensure that all data files are uploaded as 'Supporting Information' and are invariably referred to (in the manuscript, figure legends, and the Description field when uploading your files) using the following format verbatim: S1 Data, S2 Data, etc. Multiple panels of a single or even several figures can be included as multiple sheets in one excel file that is saved using exactly the following convention: S1_Data.xlsx (using an underscore).

2) Deposition in a publicly available repository. Please also provide the accession code or a reviewer link so that we may view your data before publication.

Regardless of the method selected, please ensure that you provide the individual numerical values that underlie the summary data displayed in the following figure panels as they are essential for readers to assess your analysis and to reproduce it:

Fig. 1D, F-J; Fig. 2D, F, G, J-L; Fig. 3I, J; Fig. 4D-I; Fig. 5CI, CII, H-K; Fig. 6B, D-FII; Fig. 7B, CII-EII; Fig. 8B-E, G, I-K; Fig. S1C; Fig. S2B, D; Fig. S3B-D, F and Fig. S4B, C, E

NOTE: the numerical data provided should include all replicates AND the way in which the plotted mean and errors were derived (it should not present only the mean/average values).

Please also ensure that figure legends in your manuscript include information on WHERE THE UNDERLYING DATA CAN BE FOUND, and ensure your supplemental data file/s has a legend.

Please ensure that your Data Statement in the submission system accurately describes where your data can be found.

------------------------------------------------------------------------

CODE POLICY

Per journal policy, please make the Python & Fiji scripts publicly available at this stage in Zenodo. Please ensure that the code is sufficiently well documented and reusable, and that your Data Statement in the Editorial Manager submission system accurately describes where your code can be found. More information on our Code Policy, what and how to share can be found here: https://journals.plos.org/plosbiology/s/code-availability

------------------------------------------------------------------------

Reviewers' comments

Rev. 1:

Dear Authors, thank you for your revised manuscript and your detailed response. I am pleased to confirm that all required modifications have been made and that the major concerns raised in my initial review have been adequately addressed. The manuscript meets the standard required for publication.

Rev. 2: Barbara A. Niemeyer - note that this reviewer has signed the review.

The authors have done an excellent and very comprehensive revision of the manuscript, carefully taking the raised points into consideration. The addition of a substantial number of new mechanistic experiments strengthen the already detailed manuscript further and make it an excellent reference for future research.

# Author comment: Author response to Decision Letter 3

**DOI:** 10.1371/journal.pbio.3003910.r005

29 Jun 2026

Attachment Submitted filename: Response_to_Reviewers_auresp_3.pdf

# Editor report: Decision Letter 3

**DOI:** 10.1371/journal.pbio.3003910.r006

**Authors:** Ines Alvarez-Garcia

3 Jul 2026

Dear Dr Spehr,

Thank you for the submission of your revised Research Article entitled "Cell type-specific Ca2+ signals govern mouse seminiferous tubule physiology" for publication in PLOS Biology. On behalf of my colleagues and the Academic Editor, Carmen Williams, I am delighted to let you know that we can in principle accept your manuscript for publication, provided you address any remaining formatting and reporting issues. These will be detailed in an email you should receive within 2-3 business days from our colleagues in the journal operations team; no action is required from you until then. Please note that we will not be able to formally accept your manuscript and schedule it for publication until you have completed any requested changes.

Please take a minute to log into Editorial Manager at http://www.editorialmanager.com/pbiology/, click the "Update My Information" link at the top of the page, and update your user information to ensure an efficient production process.

PRESS

We frequently collaborate with press offices. If your institution or institutions have a press office, please notify them about your upcoming paper at this point, to enable them to help maximise its impact. If the press office is planning to promote your findings, we would be grateful if they could coordinate with biologypress@plos.org. If you have previously opted in to the early version process, we ask that you notify us immediately of any press plans so that we may opt out on your behalf.

We also ask that you take this opportunity to read our Embargo Policy regarding the discussion, promotion and media coverage of work that is yet to be published by PLOS. As your manuscript is not yet published, it is bound by the conditions of our Embargo Policy. Please be aware that this policy is in place both to ensure that any press coverage of your article is fully substantiated and to provide a direct link between such coverage and the published work. For full details of our Embargo Policy, please visit http://www.plos.org/about/media-inquiries/embargo-policy/.

Many congratulations and thanks again for choosing PLOS Biology for publication and supporting Open Access publishing. We look forward to publishing your study.

Sincerely,

Ines

--

Ines Alvarez-Garcia, PhD

Senior Editor

PLOS Biology

ialvarez-garcia@plos.org
